# Spatially-targeted tuberculosis screening has limited impact beyond household contact tracing in Lima, Peru: A model-based analysis

**DOI:** 10.1371/journal.pone.0293519

**Published:** 2023-10-30

**Authors:** Joshua Havumaki, Joshua L. Warren, Jon Zelner, Nicolas A. Menzies, Roger Calderon, Carmen Contreras, Leonid Lecca, Mercedes C. Becerra, Megan Murray, Ted Cohen

**Affiliations:** 1 Department of Epidemiology of Microbial Diseases, Yale University, New Haven, CT, United States of America; 2 Department of Biostatistics, Yale School of Public Health, New Haven, CT, United States of America; 3 Department of Epidemiology, University of Michigan, Ann Arbor, MI, United States of America; 4 Center for Social Epidemiology and Population Health, University of Michigan School of Public Health, Ann Arbor, MI, United States of America; 5 Department of Global Health and Population, Harvard T. H. Chan, School of Public Health, Boston, MA, United States of America; 6 Socios en Salud Sucursal Peru, Lima, Peru; 7 Programa Acadêmico de Tuberculose, Faculdade de Medicina, Universidade Federal do Rio de Janeiro, Rio de Janeiro, Brazil; Burnet Institute, AUSTRALIA

## Abstract

Mathematical models have suggested that spatially-targeted screening interventions for tuberculosis may efficiently accelerate disease control, but empirical data supporting these findings are limited. Previous models demonstrating substantial impacts of these interventions have typically simulated large-scale screening efforts and have not attempted to capture the spatial distribution of tuberculosis in households and communities at a high resolution. Here, we calibrate an individual-based model to the locations of case notifications in one district of Lima, Peru. We estimate the incremental efficiency and impact of a spatially-targeted interventions used in combination with household contact tracing (HHCT). Our analysis reveals that HHCT is relatively efficient with a median of 40 (Interquartile Range: 31.7 to 49.9) household contacts required to be screened to detect a single case of active tuberculosis. However, HHCT has limited population impact, producing a median incidence reduction of only 3.7% (Interquartile Range: 5.8% to 1.9%) over 5 years. In comparison, spatially targeted screening (which we modeled as active case finding within high tuberculosis prevalence areas 100 *m*^2^ grid cell) is far less efficient, requiring evaluation of ≈12 times the number of individuals as HHCT to find a single individual with active tuberculosis. Furthermore, the addition of the spatially targeted screening effort produced only modest additional reductions in tuberculosis incidence over the 5 year period (≈1.3%) in tuberculosis incidence. In summary, we found that HHCT is an efficient approach for tuberculosis case finding, but has limited population impact. Other screening approaches which target areas of high tuberculosis prevalence are less efficient, and may have limited impact unless very large numbers of individuals can be screened.

## Introduction

The global incidence of tuberculosis (TB) is declining at approximately 1–2% per year [1], far too slowly to meet control targets established in the World Health Organization’s End TB Strategy [2, 3]. In most high incidence settings, TB case detection depends on individuals recognizing their symptoms and presenting for care. This passive approach to case detection means that, in some settings, there are substantial numbers of individuals with active infectious TB that have not yet sought care and may never self-present to the health system for diagnosis [4, 5]. The World Health Organization estimates that approximately 37% of incident cases each year remain undiagnosed and untreated [6]. Further, these undiagnosed individuals play an important role in transmission. [7, 8].

Active case finding (ACF) interventions aim to shorten delays to diagnosis and minimize the number of undiagnosed individuals by seeking out new cases of TB in the community. ACF has been proposed as a way to meet ambitious global TB control targets [2, 3, 9]. However, these ACF interventions require substantial investment and the evidence for the population-level impact of untargeted community-level ACF interventions has been mixed [10–12].

Although highly targeted case finding interventions such as contact tracing within the homes of newly diagnosed TB cases (household contact tracting: HHCT) are efficient approaches for finding new cases of TB [13–15], these interventions produce only modest population-level reductions in TB burden [12, 16, 17]. Empirical data for ACF interventions implemented at a larger spatial-scale (e.g., neighborhoods) is limited [18]. To address this knowledge gap, several modeling studies have estimated the potential impacts of spatially-targeted ACF interventions, concluding that focusing case-finding efforts on local areas of elevated TB burden can generate wider benefits for the community [19, 20]. Although these models incorporate some aspects of the spatial structure of *Mycobacterium tuberculosis* (Mtb) transmission, they have not generally attempted to realistically represent the spatial distribution of TB in communities or tried to compare the effects of screening interventions of realistic scale. For example, previous models of spatial ACF interventions have adopted a metapopulation approach [21] in which they assume homogeneous mixing within a small number of geographic regions which differ by population size and incidence rate and assume that a large fraction of the population (i.e., thousands of individuals) within an entire region can be screened for disease. We hypothesize that more detailed representation of the spatial distribution of TB in communities and more realistically-scaled ACF interventions are needed to better understand the efficiency and likely impact of spatially-targeted ACF interventions.

In this paper, we describe an individual-based transmission model that includes household transmission and also allows for Mtb transmission to vary by spatial proximity in the community. This model allows us to recreate observed local and global features of the spatial distribution of TB case notifications in homes and in the community of a district in Lima, Peru. We then use our calibrated model to estimate the efficiency and incremental impact of HHCT and a small-scale spatially-targeted ACF intervention implemented outside the homes of known TB cases.

## Methods

### Setting

**Fig 1:**
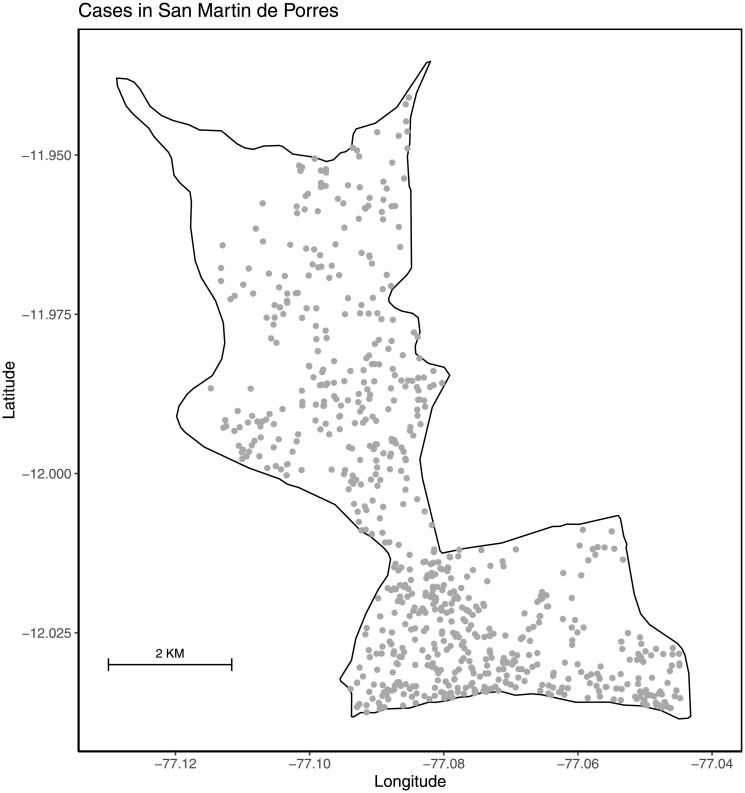
Georeferenced TB case notifications in San Martin de Porres. Grey dots are case notifications from the 33-month prospective cohort study. Case locations represent households of individuals and were jittered to ensure anonymity.

We calibrated our model to georeferenced TB case notification data representing the households of individuals from a prospective cohort study conducted in Lima, Peru between 2009 and 2012 (See [22] for details on the study). Due to the computational demands of the model, we focused on San Martin de Porres (SMP), a single district within the larger study area. SMP is an urban setting that had a population density of ≈ 17, 000 people per *KM*^2^ in 2009 [23, 24]. In Lima, and consistent with other high incidence settings, more transmission events occur in the community than in the households of individuals with active disease [25, 26]. Over the course of 33 months of study in SMP, there were a total of 799 TB case notifications out of a total population of ≈615, 800 individuals (Fig 1) [23].

### Distribution of at-risk individuals and households

**Fig 2:**
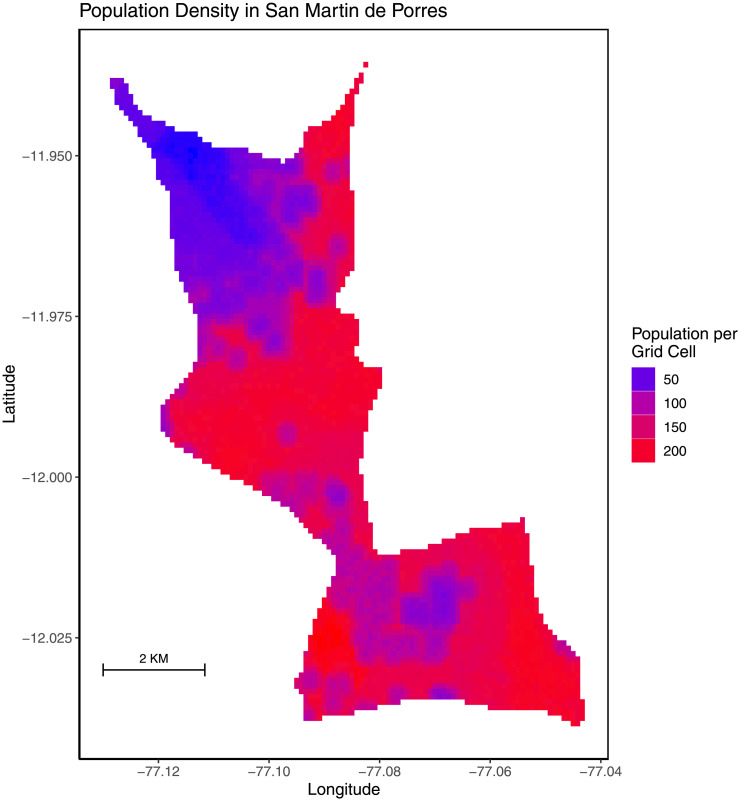
Population density of San Martin de Porres. Unadjusted *Worldpop* data from 2009 from Peru informed our model [23].

To accurately capture heterogeneity in the spatial distribution of the SMP population, we used *Worldpop* data for Peru from 2009 which provides population counts by ≈100 *m*^2^ grid cells covering the entire district [23]. Therefore, we divided our modeled district into grid cells corresponding to the *Worldpop* data. Next, for each model grid cell, we created households with membership sizes drawn from the distribution of household sizes in Peru [27] (we made assumptions about the distribution of larger household sizes i.e., those with ≥ 6 members that did not have discrete counts associated with them) until the total population in the model grid cell was approximately equal to the population in the corresponding *Worldpop* data grid cell (an average of ≈140 individuals per grid cell). We placed all households in a given grid cell near its centroid because we did not have the exact coordinates of household locations. We created 10 realizations of the population of SMP which we then used to simulate transmission. Fig 2 displays the modeled population density of SMP and Table 1 provides parameter values which informed our model initialization. Additional modeling details and comparisons between observed and modeled household size distribution are provided in S1 Section in S1 Appendix.

**Table 1:** Parameters and uncertainty ranges.

Parameter	Description (units)	Range	Source/Explanation
Household size distribution		1 to 9	Membership sizes were sampled from the distribution of household sizes in Peru in 2007 [27]. We assumed that larger households (that didn’t have discrete counts associated with them) followed a triangular distribution (S1 Section in S1 Appendix).
Number of Seeds	Number of individuals starting in *I*	20 to 150	Bounds were set empirically to reduce stochastic extinction and speed up time to steady state.
Seed range	The area within which infectious individuals are seeded (KM)	0.15 to 1.5	Bounds were set empirically to obtain the target range of case notifications and help recreate the spatial distribution of the data.
*β*_*HH*_	Per-capita household transmission parameter (infections/month)	0.17 to 1	Bounds were set empirically to obtain the target range of case notifications. Derived by sampling values for the unscaled transmission parameter, *β*_*uHH*_ (range: 0.5 to 3) and dividing by the mean number of household contacts (S2 Section in S1 Appendix).
*β*_*C*_	Per-capita community transmission parameter (infections/month)	1.3*e*^−07^ to 0.015	Bounds were set empirically to obtain the target range of case notifications. *β*_*scalar*_ values were sampled (range: 0.1 to 0.7), multiplied by *β*_*uHH*_ and then transformed by the number of community contacts (S2 Section in S1 Appendix).
*θ*	Spatial range of community contacts (KM)	0 to 5.47	Upper bound set from [29]
*α*	Power term used in the transmission kernel (Eq 1)	0 to 0.7	Bounds were set empirically to obtain the target range of case notifications.
*ω*	Amount of immunity conferred by current state (%)	For *S* = 0%; *EL*, *LL*, and *R* = 80%; *I*, *T* = 100%	[32, 33]
*μ*	Mortality rate (*years*^−1^)	1/74	Life expectancy in Peru in 2009 [34]
*ϵ*	Early latency progression rates (/yr)	*EL*_1_: 0.04 to 0.07 *EL*_2_: 0.02 to 0.03 *EL*_3_: 0.007 to 0.01 *EL*_4_: 0.003 to 0.005 *EL*_5_: 0.001 to 0.002	Fitted model parameter values from [35] ±25%
*τ*	Late latency progression rate (/yr)	0.0006 to 0.001	Fitted model parameter values from [35] ±25%
*γ*	Recovery rate (/yr)	0.09 to 0.15	[16, 36]
*κ*	Active TB mortality rate (/yr)	0.05 to 0.4	[16, 36]
ATB treat	Treatment rate (/yr)	2.1 to 6.3	All detected individuals were given treatment. The case detection rate was sampled (range: 80% ±10% [37]) and the treatment rate was calculated accounting for competing risks (S4 Section in S1 Appendix).
coverage	Number screened for each high prevalence grid cell	20 to 100	A number of individuals (e.g., 20 individuals) are screened for each detected index case.
txd	Treatment duration (months)	6	[38]

### Model of TB natural history and interventions

**Fig 3:**
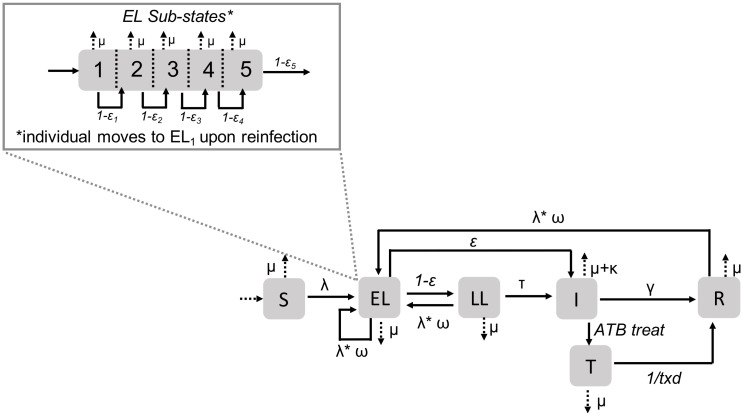
Mtb transmission model schematic. The 5 disease states included in our model are susceptible (*S*), early latent (*EL*), late latent (*LL*), infectious TB (*I*) and recovered (*R*), and the intervention state is Treatment (*T*). The parameters are as follows, *μ* is the mortality rate, λ is the force of infection (see Eq 1 for details) which is modulated by *ω* to account for partial immunity among previously or currently infected individuals, *ϵ* is the rate of progression from early latent to active TB (rates of progression decrease as time since infection increases as represented in the *EL* sub-states panel), *τ* is the rate of progression from late latent to active TB, *κ* is the active TB mortality rate, *γ* is the rate of spontaneous recovery from active TB, *ATB* *treat* is the treatment rate of individuals in active TB, and finally *txd* is the duration of treatment. Births and deaths are represented by dotted lines.

We extended a previously published individual-based Mtb transmission model [28] which was based on [16] and includes coupled community and household transmission. The natural history model includes five distinct TB disease states: susceptible (*S*), early latent (*EL*), late latent (*LL*), infectious active TB (*I*), and recovered (*R*) and one intervention state: treatment (*T*) (Fig 3).

In our model, susceptible (*S*), latent (*EL* and *LL*), and recovered (*R*) individuals are infected according to the force of infection:

$$\begin{array}{c} \begin{array}{cc} \lambda_{HHj}\left(t\right) & =\beta_{HH}\sum_{i=1}^{Pop_{HHj}-1}1\left\{state_{i}\left(t\right)==I\right\}\\ \lambda_{Cj}\left(t\right) & =\beta_{C}\sum_{i=1}^{Pop_{Cj}-1}\frac{1\left\{state_{i}\left(t\right)==I\right\}\left\{|\right|L_{i}-L_{j}|\left|\leq \theta \right\}}{\left|\right|L_{i}-L_{j}{\left|\right|}^{\alpha }}\\ \lambda_{j}\left(t\right) & =\left\{\lambda_{HHj}\left(t\right)+\lambda_{Cj}\left(t\right)\right\}\omega \left\{state_{j}\left(t\right)\right\} \end{array} \end{array}$$ (1)

where the force of infection exerted upon individual *j* at time *t* is the sum of household and community transmission. The household transmission parameter, *β*_*HH*_, is multiplied by the total number of infectious household contacts of *j*. ‘1{.}’ is an indicator function which equals 1 if a given criteria is met and 0, if not. Therefore, we check the state of each household contact (the total number of contacts corresponds to the number of individuals in the household minus 1 (*Pop*_*HHj*_ − 1)), and count the number that currently have active TB (*I*). Next, the community transmission parameter, *β*_*C*_, is multiplied by the total number of infectious community contacts of *j* and modified by a power transmission kernel as described below.

We calculate the haversine distance (||*L*_*i*_ − *L*_*j*_||) between *j* and each individual in the community with active TB to determine if they are within the spatial range (*θ*). An infectious community contact of *j* must have active TB and be within the specified distance. Within the community, the transmission intensity of each contact is transformed by a power transmission kernel (in which distance is raised to the power, *α*) representing human mobility patterns in which intensity decays quickly over short distances, but areas that are far apart maintain a small amount of spatial interaction [29–31]. To prevent very large values of the community transmission parameter when the distance is small (i.e., among individuals in the same centroid), we assumed that all individuals within a given centroid were separated by 50 meters which is approximately half of the distance between adjacent centroids. Finally, the force of infection for the household and community are summed and modified by *ω*(*state*_*j*_(*t*)), to account for partial immunity (in which *ω*(*state*_*j*_(*t*)) is set to 0 for susceptible individuals), conferred by a previous infection [32, 33]. See S2 and S3 Sections in S1 Appendix for more details on the transmission model and Table 1 all model parameters and their ranges.

### Distribution of TB cases and calibration of model

Our goal in model calibration was to generate epidemic simulations in which the spatial distribution of TB in the model matched the observed local case intensity while maintaining features of the overall spatial structure of TB in the community (see Fig 1 for mapped case notifications). Parameter sets were deemed better matches to the data when they recreated regions of high (or low) intensity transmission that were in close spatial proximity to corresponding high (or low) intensity transmission regions in the data. We then compared a measure of global autocorrelation between the modeled simulations and the data.

We used a sample-importance-resampling [39] approach to select parameter sets that calibrated well to the observed data. In the *sample* stage, we proposed 10,000 vectors of parameters, with each parameter selected at random from its prior range and we ran the model to steady state. To determine how well each simulation calibrates to the data (the *importance* stage), we calculated kernel density estimates (KDEs) of the 2-dimensional (i.e., latitude and longitude) spatial distribution of case notifications from each model run and compared these to the KDE of the observed data. We assumed that upon detection, individuals with active TB were given treatment, therefore case notifications corresponded to individuals that transitioned to the treatment state. KDEs provide a smoothed estimate of the underlying probability density function such that the density estimator of the KDE is higher in regions with more cases and lower in regions with fewer cases [40, 41]. We compared the model and data KDEs using the Kullback-Leibler divergence (KLD) [42] which quantifies how much information would be lost if we were to approximate the data KDE with the model KDE. Lower KLD values therefore represent better matches between the model and data. Finally, in the *resampling* stage, we sampled 2,500 parameter sets with replacement using the inverse of the KLD as sampling weights. While this calibration approach primarily focuses on matching localized spatial distribution of TB case notifications, we also examined two additional global metrics among resampled parameter sets. First, we determined if the model performs better than random using a permutation test and second, we compared the global spatial autocorrelation to the data among each resampled parameter set using Global Moran’s I (see S8 Section in S1 Appendix for details) [43, 44].

We investigated several additional calibration approaches and provide details of these in S5 Section in S1 Appendix. The major findings on the impact and efficiency of spatially targeted ACF we present in the main text are not sensitive to the calibration approach we chose. Analyses were conducted in R version 3.4.1 [45]. We calculated the KDEs using the ‘kde’ command in the ks package [46] and the KLD using the ‘kl.dist’ command in the seewave package [47].

### Active case finding intervention scenarios

The models were calibrated under the assumption that passive case finding will always be available, such that individuals with active TB (*I*) can self-present for diagnosis. Once diagnosed, we assume that treatment (*T*) immediately eliminates infectiousness and individuals ultimately recover to *R*.

All active case finding (ACF) interventions were introduced after model calibration (i.e., after the simulated study period). The ACF interventions we consider are triggered by the passive detection of an individual with active TB (i.e., an “index case”). For simplicity, we assume that active screening occurs simultaneously with the detection of the index case, though in reality this active screening would occur after. We also assume 100% diagnostic accuracy of our screening interventions and full compliance with treatment, which likely lead to overestimates of the benefits of such screening programs.

#### Spatially targeted ACF intervention

We simulate High Prevalence Grid Cell (HPGC) screening in which for each index case detected, 20 to 100 individuals are randomly selected and screened from a cell. Cells are sampled using the period prevalence from the model calibration as weights. In other words, if there are more cases in a given cell before the intervention, it is more likely to be targeted. We use this weighting scheme, because we want to explore a best-case scenario under reasonable circumstances and expect this to result in more efficient detection of undiagnosed cases.

**Fig 4:**
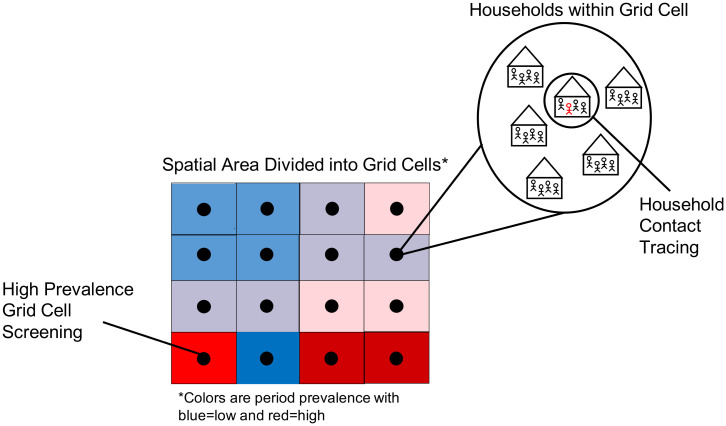
Active case finding interventions. For a given detected index case (individual in red): ‘Household Contact Tracing’ screens all household contacts, ‘High Prevalence Grid Cell Screening’ screens 20 to 100 individuals in a cell that is selected based on the period prevalence. In this schematic, the cells are colored such that higher intensity transmission areas are in red and lower transmission intensity areas are in blue.

We speculated that screening 20 to 100 individuals for each detected index case represents a reasonable amount of screening effort that could be done by a small team in short time period (i.e., over a few days). On average, this corresponded to between 14% and 71% of the population of an entire cell. If the number needed to screen was larger than the population of the cell then we assumed that all individuals in that cell were screened. We examined the effects of HHCT alone and also, HHCT in conjunction with HPGC screening (see Fig 4).

### Assessing the performance of interventions

We ran each intervention for a 5-year duration on each resampled parameter set. We first compared the efficiency of each intervention scenario over the entire 5-year period by dividing the total numbers of individuals screened by the total number of previously undetected, found cases of active TB. Next, to compare the overall protective impacts of different interventions, we calculated rate ratios (RRs) comparing the incidence rate over the entire 5-year period for each intervention to the counterfactual scenario where there was only a continuation of passive case finding. The incidence rate includes all (detected and undetected) individuals who progress to active TB (*I*).

### Sensitivity analysis

To test the sensitivity of our results to our calibration approach, we first re-ran interventions on 5,000 (instead of 2,500) resampled parameter sets. Next, we ran the interventions on resampled parameter sets selected based upon an alternative calibration method. This alternative method involved comparing model outputs to the data by groups of centroids. We compared across groups of centroids to account for spatial proximity. Specifically, for a given centroid, we summed (1) all cases in the centroid, (2) all cases in grid cells adjacent to the centroid (using a ‘queen’ definition), and (3) all cases in the adjacent grid cells of the adjacent grid cells. We repeated this until 5^*th*^ degree neighbors were obtained (denoted the ‘5^*th*^ degree neighbors method’). We then took ∣*I*_*data*_ − *I*_*model*_∣ for each group and summed over the entire district. We could not form groups higher than the 5^*th*^ degree due to limitations in computational power. However, this corresponded to a radius of 0.5 kilometers which we assumed to be sufficient for smoothing. While the KDE method smooths the case counts across centroids, this 5^*th*^ degree neighbors method separates centroids into discrete groups and therefore accounts for spatial proximity differently.

Next, to test the sensitivity of our results to different key parameter values, we examined how the efficiency and impacts of different intervention scenarios change when the values of key model parameters representing transmission intensity over space are varied (e.g., spatial range, community transmission rate etc.).

Finally, to examine key assumptions related to our interventions, we conducted additional sensitivity analyses. First, we reran all interventions assuming a diagnostic sensitivity of 70% (instead of the previously assumed 100%). We chose this value to correspond to the performance of symptom screening which is the most common method of screening. [48]. Second, we changed our HPGC characterization method by using case notifications over the study period instead of period prevalence. In other words, cells with higher case notifications were more likely to be targeted. Third, for the HPGC screening intervention, we scaled up the screening area to be blocks of 4 and 16 grid cells. Fourth, we examined the effects of increasing the number of individuals screened scaled by the size of the screening area e.g., we screened 4 times 20–100 when the screening area consisted of blocks of 4 grid cells.

## Results

### Calibration results

Our calibration methods selected for parameter sets that best recreated the observed local case intensity data. See S4 Fig in S1 Appendix for comparison between our best calibrating parameter set and the observed data. We additionally examined how well our resampled parameter sets recreated the global spatial structure of the data. Results from our permutation test revealed that parameter sets for simulations with lower KLD values (i.e., those that produced simulations which better matched data) performed better than the majority of random permutations to the model output (S11 Fig in S1 Appendix). Finally, comparison of Moran’s I revealed that global spatial autocorrelation among resampled parameter sets matched the data well with an R-squared value of 0.95 (see S12 Fig (S1 Appendix) for results and S5-S8 Figs (S1 Appendix) for epidemiological features of our modeled population).

### Performance of interventions

#### Efficiency

**Fig 5:**
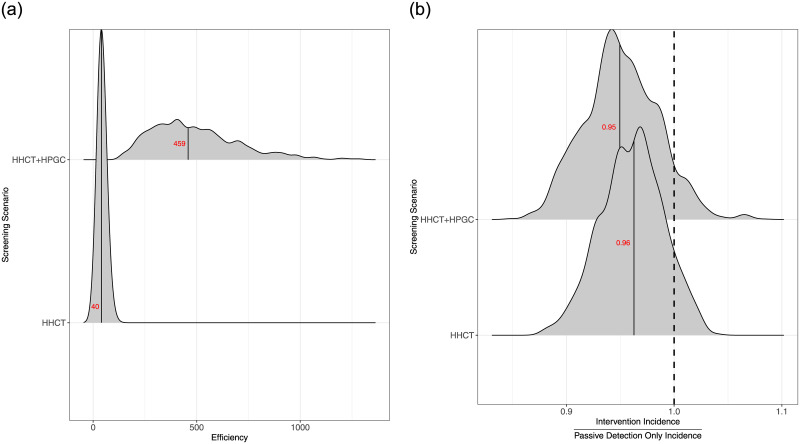
Ridgeline plots displaying the efficiency and impact of interventions. (a) The efficiency of different screening interventions. ‘Efficiency’ was defined as the number of individuals that need to be screened to find a case of active TB and (b) Rate ratios comparing the 5-year incidence rate of different screening interventions to passive surveillance only. Medians are in red.

**Fig 6:**
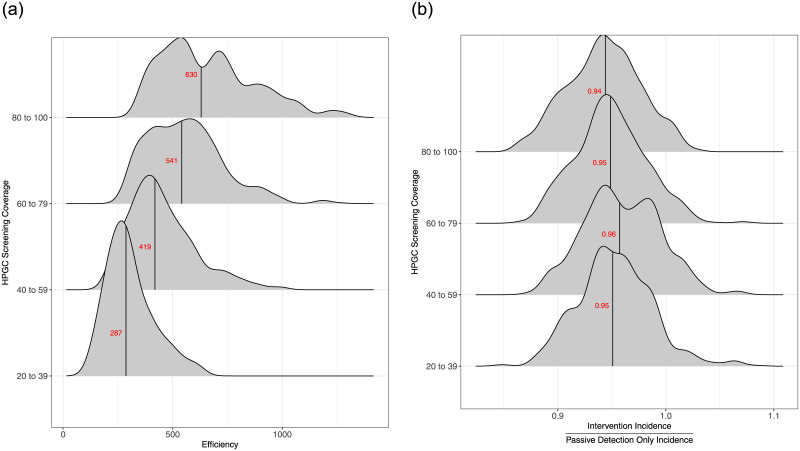
Ridgeline plots displaying (a) The efficiency of HPGC screening stratified by coverage. ‘Efficiency’ was defined as the number of individuals that need to be screened to find a case of active TB and (b) Rate ratios comparing the 5-year incidence rate of HPGC to passive surveillance only stratified by coverage. Medians are in red.

As expected, HHCT was a highly efficient intervention with a median of 40 (Interquartile Range (IQR): 31.7 to 49.9) household contacts required to be screened to detect a single previously undetected case of active TB (Fig 5a). HPGC in conjunction with HHCT required the evaluation of ≈ 12 times more individuals to find a single case of active TB. Further, efficiency decreases as number of individuals screened increases in the HPGC screening intervention (Fig 6a).

#### Impacts

HHCT alone resulted in a median 3.7% (IQR: 5.8% to 1.9%) reduction in TB incidence over the course of the 5-year intervention compared to a counterfactual scenario with passive case finding only (Fig 5b). The addition of HPGC screening resulted in limited incremental benefit, with a median total expected incidence reduction of 5.1% (IQR: 7% to 2.6%). There is a modest increase in impact as number of individuals screened increases in the HPGC screening intervention (Fig 6b).

### Sensitivity analysis

To confirm the robustness of our results to our calibration methods, we first ran interventions on 5,000 resampled parameter sets (instead of 2,500) and found that overall, results were consistent (see S13 Fig in S1 Appendix). Next, to confirm the robustness of our results to the method in which calibration accounted for spatial proximity, we resampled parameter sets selected using our 5^th^ degree neighbors method (described above). We found that in this analysis, the relative efficiency and impact of different intervention scenarios were similar to those presented here (see S14 Fig in S1 Appendix for details).

Next, to test the sensitivity of our results to key parameter values, we examined how the impact and efficiency of intervention scenarios change in response to altering the intensity of transmission over space (see S15 and S16 Figs in S1 Appendix for details). Overall, the relative performance of interventions was consistent across different values of the household transmission rate, community transmission rate, spatial range and the power term (in the power transmission kernel).

Finally, to test the robustness of our results to assumptions about our intervention scenarios, we conducted additional sensitivity analyses. First, we examined the performance of interventions assuming a diagnostic sensitivity of 70% (S17 Fig in S1 Appendix). Overall, we found that the impact was slightly decreased, but the relative performance of interventions were robust to a lower diagnostic sensitivity. Second, we weighted cells by total number of case notifications over the simulated study period (instead of using period prevalence) for the HPGC screening intervention (S18 Fig in S1 Appendix). Overall, performance of HPGC did not change. Finally, we scaled up the screening area and the number of individuals screened for the HPGC screening intervention (S19 and S20 Figs in S1 Appendix). Overall, we found no effect on impact and a slight decrease in efficiency as the screening area increases. On the other hand, as the number of individuals screened increases, the impact of increases. However, consistent with previous results, requiring the screening of more individuals leads to decreased efficiency.

## Discussion

Our analyses support investment in HHCT which is a highly efficient approach for finding new cases of active TB; in the SMP district of Lima, we estimate that a median of 40 (IQR: 31.7 to 49.9) household contacts must be screened to detect a new case. However, although HHCT is highly efficient, this intervention alone confers only modest population-level impact with a median TB incidence reduction of 3.7% (IQR: 5.8% to 1.9%) above passive case finding alone over the course of 5 years (Fig 5). In our model, we find that adding HPGC screening to HHCT provides only small incremental benefits in reducing TB incidence, and requires large increases in screening capacity to generate these benefits.

Results presented here are consistent with observational studies that have found HHCT to be an efficient means of ACF. A community-based study in South Africa found that clustering of TB in households leads to HHCT being more efficient than untargeted screening [15]. Modeling studies have also found similar results. Zelner et al. used data from this same cohort study in Lima and found that administering preventive therapy to household contacts with latent TB prevents more cases than a untargeted community-based approach [13]. Due to its efficiency, HHCT is recommended in low resource settings [49], however, aside from a few exceptions, it is not regularly conducted [50]. Our results support additional programmatic investment in HHCT as it is an efficient means for identifying individuals at greatest risk for TB.

Empirical studies on spatially-targeted ACF have shown inconsistent population-level results for TB as well as for other infectious diseases. A recent review found no evidence that targeting areas of higher malaria transmission (i.e., hotspots) with malaria interventions accelerates its elimination [51]. While studies on TB in low incidence settings have found that spatial and molecular data can be used to efficiently find new cases [52, 53], there is little empirical data from higher burden settings like Lima [18] to support this approach. To address this knowledge gap, modeling studies have estimated the impact of spatially targeted interventions in high burden settings. Dowdy et al. found that targeting hotspots could have large benefits for TB control in Rio De Janeiro [19]. The much smaller impact estimated in our analysis in Lima is a result of differences in both the relative scale of our ACF interventions and the way in which we attempted to capture local heterogeneity in the distribution of TB in our model. Our HPGC intervention screened relatively few individuals (i.e., 20 to 100 for each detected index case) compared with Dowdy et al. in which screening was assumed to include hundreds of thousands of individuals. Further, as we scaled up the number of individuals screened in the HPGC intervention, we were able to confer slight increases in impact. Importantly however, this led to a decreases in efficiency (Fig 6). To confer substantial increases in impact, we needed to increase both the screening area and the number of individuals screened (S20 Fig in S1 Appendix), but again this improvement in impact came at the cost of efficiency. We also attempted to capture realistic local patterns of TB notifications in our model, which typically include multiple small loci of increased disease burden, while Dowdy et al. used a compartmental modeling approach with a single region of high transmission; if indeed it is feasible to cover entire geographic regions with ACF screening interventions, the larger impacts estimated by Dowdy et al. may be more easily obtained. Overall, our spatially-targeted interventions rely on the assumption that local transmission is a key driver of tuberculosis incidence, however, we still found that the impact of the HPGC interventions was limited. Consistent with this, a recent paper used whole genome sequence data and spatial locations of TB cases from the same study in Lima, Peru, and found that local incidence does not correlate well with local transmission [54].

Our findings highlight how challenging it is to identify TB ACF interventions which are both efficient and impactful. It is abundantly clear that HHCT must be a priority for programs, but this will not be sufficient to meet aggressive targets for TB control. ACF must also be implemented outside the homes of known TB cases, but our findings suggest that in areas with epidemiology similar to our setting, spatially-targeted strategies may neither be sufficiently efficient nor effective to justify their widespread use.

So, how to proceed? New strategies for ACF must be able to readily identify relatively large groups of individuals who are at high risk of being recently infected with TB (and thus at risk of rapid progression to TB) [35]. In many settings, individuals that are incarcerated are at extremely high risk of recent TB infection [55, 56] and increased screening in prisons and jails, especially in countries where the sizes of these at-risk populations are large, is likely to be an attractive ACF strategy [57]. Individuals with known characteristics associated with elevated incidence of TB, such as a history of prior TB treatment [58, 59] or immunosuppression [60], are obvious choices as targets of increased screening and possibly preventive treatment, but the size of these at risk populations differ dramatically between epidemic settings and the impact of such targeting will be variable.

More speculatively, it is possible that more widespread availability of genomic sequencing can eventually lead to public health interventions that are more responsive to ongoing local epidemics of Mtb transmission. The development of biomarkers for incipient TB may eventually also help target interventions to those at highest proximal risk [61]. However, we note that these new tools, while promising for increasing efficiency of screening programs, may struggle to identify enough individuals quickly enough to dramatically bend the incidence curve.

We needed to make several important decisions as we developed approaches for calibrating our model to the spatial distribution of TB in Lima. The key driver of the spatial distribution of cases in our model was population density that was informed by *Worldpop* data [23]. We note that this gridded population data has limitations especially in low resource settings and that its accuracy is affected by many factors such as the resolution of the census data that informs it, the approach for distributing population across grid cells and the fact that smaller grid cells may have more uncertainty associated with them [62]. Additionally, our simplifying assumption that all households in a given grid cell were located near the centroid and that distances between them were 50 meters had minimal impact on the model outcomes due to the small size of each grid cell. Furthermore, imputing a distance value of 50 meters between households avoids unrealistically inflating the community transmission parameter i.e., for houses that would placed very close together in a random or alternative parameterization. Furthermore, because the population density did not correspond perfectly with the spatial distribution of case notifications, the model did not completely recreate the data. Allowing additional regional level flexibility in our model by varying transmission rates by centroid, would have generated better matches with data, but at a cost of substantial additional model complexity. We also note that we did not account for spatial heterogeneity in the case detection rate (e.g., as was addressed here [63]) and just assumed that proportion of undetected cases was homogeneous throughout SMP. Accounting for this variability would have altered the overall spatial distribution of TB in the model and could have changed how well the model calibrates to the data as well as the performance of the interventions. In the interest of parsimony, we aimed to develop a model approach which could recreate the variation in local cases and overall spatial autocorrelation of cases observed in SMP without adding in detailed regional variability in e.g., demography or healthcare access. Other modeling approaches rely on averaging over localized areas (e.g., kriging) [64] or utilize additional environmental covariates [65].

Our model also does not attempt to accurately represent a number of factors that may influence transmission and social mixing patterns like age, gender and changing household compositions and changing population density. In the absence of any other data, the value for the spatial range of community contacts was taken from a paper on tuberculosis spillover from a prison population [29]. Although this likely would not be the same among individuals living in the community, incorporating longer distance contacts would likely have minimal impacts on the model dynamics. Furthermore, we made simplifying assumptions about the replacement of individuals who die (see S3 Section in S1 Appendix). However, the objective of our analysis was to focus on drivers of the spatial distribution of TB and project interventions over short time scales (i.e. 5 years). Therefore, we assumed that demographic and epidemiological characteristics would be relatively stable and only incorporated factors that we believed would have direct impact on the spatial distribution of cases e.g. population density. We did run interventions over a longer (10-year) timeframe and observed an increase in impact (S21 Fig in S1 Appendix), but these simulations did not incorporate changing demographic and population characteristics that would be important to include over longer time scales. We note that the interaction between these factors (e.g., age, transmission and the spatial distribution of cases) over longer time scales would be useful to explore in future modeling work.

In summary, we found that HHCT is an efficient ACF approach, but is unlikely to generate large reductions in TB incidence. In areas with epidemiology similar to our setting in Lima, spatially-targeted interventions may be neither sufficiently efficient nor sufficiently effective in reducing TB incidence to justify their use. More work is needed to explore whether detailed and changing demography and epidemiology over longer time scales, or other complementary interventions like preventive therapy for individuals with latent tuberculosis may alter the impact or efficiency of spatially targeted interventions. In order to accelerate declines in TB incidence, we must find better ways to identify large numbers of individuals with early undetected TB or with elevated risk of incipient disease.

## Supporting information

S1 Appendix Supporting information. Supporting information includes household size distribution, further details on transmission model states and parameters, further details on calibration methods and results, resampled parameter value distributions, alternative calibration methods and intervention result based on different sensitivity analyses.
(PDF)

## References

[1] World Health Organization, et al. Global tuberculosis report 2019. Geneva: WHO. 2019;.

[2] Uplekar M, Weil D, Lonnroth K, Jaramillo E, Lienhardt C, Dias HM, et al. WHO’s new end TB strategy. The Lancet. 2015;385(9979):1799–1801. doi: 10.1016/S0140-6736(15)60570-0 25814376

[3] Organization WH, et al. Draft global strategy and targets for tuberculosis prevention, care and control after 2015. World Health Assembly Geneva: WHO. 2014;.

[4] Baussano I, Bugiani M, Gregori D, Van Hest R, Borraccino A, Raso R, et al. Undetected burden of tuberculosis in a low-prevalence area. The International Journal of Tuberculosis and Lung Disease. 2006;10(4):415–421. 16602406

[5] Guwatudde D, Zalwango S, Kamya MR, Debanne SM, Diaz MI, Okwera A, et al. Burden of tuberculosis in Kampala, Uganda. Bulletin of the World Health Organization. 2003;81:799–805. 14758406 PMC2572356

[6] Organization WH, et al. Global tuberculosis report 2015: World Health Organization. Geneva. 2015;.

[7] Cheng S, Chen W, Yang Y, Chu P, Liu X, Zhao M, et al. Effect of diagnostic and treatment delay on the risk of tuberculosis transmission in Shenzhen, China: an observational cohort study, 1993–2010. PLoS One. 2013;8(6):e67516. doi: 10.1371/journal.pone.0067516 23826313 PMC3694886

[8] Harris TG, Meissner JS, Proops D. Delay in diagnosis leading to nosocomial transmission of tuberculosis at a New York City health care facility. American journal of infection control. 2013;41(2):155–160. doi: 10.1016/j.ajic.2012.02.015 22750037

[9] Getahun H, Raviglione M. Active case-finding for TB in the community: time to act. The Lancet. 2010;376(9748):1205–1206. doi: 10.1016/S0140-6736(10)61503-6 20923714

[10] Marks GB, Nguyen NV, Nguyen PT, Nguyen TA, Nguyen HB, Tran KH, et al. Community-wide Screening for Tuberculosis in a High-Prevalence Setting. New England Journal of Medicine. 2019;381(14):1347–1357. doi: 10.1056/NEJMoa1902129 31577876

[11] Corbett EL, Bandason T, Duong T, Dauya E, Makamure B, Churchyard GJ, et al. Comparison of two active case-finding strategies for community-based diagnosis of symptomatic smear-positive tuberculosis and control of infectious tuberculosis in Harare, Zimbabwe (DETECTB): a cluster-randomised trial. The Lancet. 2010;376(9748):1244–1253. doi: 10.1016/S0140-6736(10)61425-0 20923715 PMC2956882

[12] Ayles H, Muyoyeta M, Du Toit E, Schaap A, Floyd S, Simwinga M, et al. Effect of household and community interventions on the burden of tuberculosis in southern Africa: the ZAMSTAR community-randomised trial. The Lancet. 2013;382(9899):1183–1194. doi: 10.1016/S0140-6736(13)61131-9 23915882

[13] Zelner J, Murray M, Becerra M, Galea J, Lecca L, Calderon R, et al. Protective effects of household-based TB interventions are robust to neighbourhood-level variation in exposure risk in Lima, Peru: a model-based analysis. International journal of epidemiology. 2017;. 10.1093/ije/dyx171 29025111

[14] Little KM, Msandiwa R, Martinson N, Golub J, Chaisson R, Dowdy D. Yield of household contact tracing for tuberculosis in rural South Africa. BMC infectious diseases. 2018;18(1):299. doi: 10.1186/s12879-018-3193-7 29973140 PMC6030742

[15] Shapiro AE, Variava E, Rakgokong MH, Moodley N, Luke B, Salimi S, et al. Community-based targeted case finding for tuberculosis and HIV in household contacts of patients with tuberculosis in South Africa. American journal of respiratory and critical care medicine. 2012;185(10):1110–1116. doi: 10.1164/rccm.201111-1941OC 22427532 PMC5448579

[16] Kasaie P, Andrews JR, Kelton WD, Dowdy DW. Timing of tuberculosis transmission and the impact of household contact tracing. An agent-based simulation model. American journal of respiratory and critical care medicine. 2014;189(7):845–852. doi: 10.1164/rccm.201310-1846OC 24559425

[17] Cavalcante S, Durovni B, Barnes GL, Souza F, Silva R, Barroso P, et al. Community-randomized trial of enhanced DOTS for tuberculosis control in Rio de Janeiro, Brazil. The international journal of tuberculosis and lung disease. 2010;14(2):203–209. 20074412 PMC3812056

[18] Cudahy PG, Andrews JR, Bilinski A, Dowdy DW, Mathema B, Menzies NA, et al. Spatially targeted screening to reduce tuberculosis transmission in high-incidence settings. The Lancet Infectious Diseases. 2019;19(3):e89–e95. doi: 10.1016/S1473-3099(18)30443-2 30554997 PMC6401264

[19] Dowdy DW, Golub JE, Chaisson RE, Saraceni V. Heterogeneity in tuberculosis transmission and the role of geographic hotspots in propagating epidemics. Proceedings of the National Academy of Sciences. 2012;109(24):9557–9562. doi: 10.1073/pnas.1203517109 22645356 PMC3386125

[20] Shaweno D, Trauer JM, Denholm JT, McBryde ES. The role of geospatial hotspots in the spatial spread of tuberculosis in rural Ethiopia: a mathematical model. Royal Society open science. 2018;5(9):180887. doi: 10.1098/rsos.180887 30839742 PMC6170575

[21] Hickson RI, Mercer GN, Lokuge KM. A metapopulation model of tuberculosis transmission with a case study from high to low burden areas. PLoS One. 2012;7(4):e34411. doi: 10.1371/journal.pone.0034411 22496801 PMC3319591

[22] Zelner JL, Murray MB, Becerra MC, Galea J, Lecca L, Calderon R, et al. Age-specific risks of tuberculosis infection from household and community exposures and opportunities for interventions in a high-burden setting. American journal of epidemiology. 2014;180(8):853–861. doi: 10.1093/aje/kwu192 25190676 PMC4188339

[23] (www worldpop org School of Geography W, Environmental Science UoSDoG, Geosciences UdN University of Louisville; Departement de Geographie, Center for International Earth Science Information Network (CIESIN) CUGHRPDPFbTB, (OPP1134076) MGF. The spatial distribution of population in 2009, Peru; 2018. Available from: 10.5258/SOTON/WP00645.

[24] Areas GA. GADM database of global administrative areas. Global Administrative Areas. 2012;.

[25] Brooks-Pollock E, Becerra MC, Goldstein E, Cohen T, Murray MB. Epidemiologic inference from the distribution of tuberculosis cases in households in Lima, Peru. Journal of Infectious Diseases. 2011;203(11):1582–1589. doi: 10.1093/infdis/jir162 21592987 PMC3096792

[26] Horna-Campos OJ, Sánchez-Pérez HJ, Sánchez I, Bedoya A, Martín M. Public transportation and pulmonary tuberculosis, Lima, Peru. Emerging infectious diseases. 2007;13(10):1491. doi: 10.3201/eid1310.060793 18257992 PMC2851510

[27] Peru 2007 Households by age and sex of reference person and by size of household.;. http://data.un.org/Data.aspx?d=POP&f=tableCode:50;.

[28] Havumaki J, Cohen T, Zhai C, Miller JC, Guikema SD, Eisenberg MC, et al. Protective impacts of household-based tuberculosis contact tracing are robust across endemic incidence levels and community contact patterns. PLoS computational biology. 2021;17(2):e1008713. doi: 10.1371/journal.pcbi.1008713 33556077 PMC7895355

[29] Warren JL, Grandjean L, Moore DA, Lithgow A, Coronel J, Sheen P, et al. Investigating spillover of multidrug-resistant tuberculosis from a prison: a spatial and molecular epidemiological analysis. BMC medicine. 2018;16(1):122. doi: 10.1186/s12916-018-1111-x 30071850 PMC6091024

[30] Albert R, Barabási AL. Statistical mechanics of complex networks. Reviews of modern physics. 2002;74(1):47. doi: 10.1103/RevModPhys.74.47

[31] Brockmann D, Hufnagel L, Geisel T. The scaling laws of human travel. Nature. 2006;439(7075):462–465. doi: 10.1038/nature04292 16437114

[32] Woldehanna S, Volmink J. Treatment of latent tuberculosis infection in HIV infected persons. The Cochrane database of systematic reviews. 2004;(1):CD000171–CD000171. 14973947 10.1002/14651858.CD000171.pub2

[33] Andrews JR, Noubary F, Walensky RP, Cerda R, Losina E, Horsburgh CR. Risk of progression to active tuberculosis following reinfection with Mycobacterium tuberculosis. Clinical infectious diseases. 2012;54(6):784–791. doi: 10.1093/cid/cir951 22267721 PMC3284215

[34] World Bank;. https://data.worldbank.org/indicator/SP.DYN.LE00.IN?end=2012&locations=PE&start=2009;.

[35] Menzies NA, Wolf E, Connors D, Bellerose M, Sbarra AN, Cohen T, et al. Progression from latent infection to active disease in dynamic tuberculosis transmission models: a systematic review of the validity of modelling assumptions. The Lancet Infectious Diseases. 2018;18(8):e228–e238. doi: 10.1016/S1473-3099(18)30134-8 29653698 PMC6070419

[36] Tiemersma EW, van der Werf MJ, Borgdorff MW, Williams BG, Nagelkerke NJ. Natural history of tuberculosis: duration and fatality of untreated pulmonary tuberculosis in HIV negative patients: a systematic review. PloS one. 2011;6(4):e17601. doi: 10.1371/journal.pone.0017601 21483732 PMC3070694

[37] WHO TB burden estimates;. https://www.who.int/teams/global-tuberculosis-programme/data.

[38] Maher D, Chaulet P, Spinaci S, Harries A, et al. Treatment of tuberculosis: guidelines for national programmes. Treatment of tuberculosis: guidelines for national programmes Second edition. 1997;(Ed. 2):1–77.

[39] Rubin DB. Using the SIR algorithm to simulate posterior distributions. Bayesian statistics. 1988;3:395–402.

[40] Chen YC. A tutorial on kernel density estimation and recent advances. Biostatistics & Epidemiology. 2017;1(1):161–187. doi: 10.1080/24709360.2017.1396742

[41] Deng H, Wickham H. Density estimation in R. Electronic publication. 2011;.

[42] Kullback S, Leibler RA. On information and sufficiency. The annals of mathematical statistics. 1951;22(1):79–86. doi: 10.1214/aoms/1177729694

[43] Moran PA. Notes on continuous stochastic phenomena. Biometrika. 1950;37(1/2):17–23. doi: 10.1093/biomet/37.1-2.17 15420245

[44] Li H, Calder CA, Cressie N. Beyond Moran’s I: testing for spatial dependence based on the spatial autoregressive model. Geographical Analysis. 2007;39(4):357–375. doi: 10.1111/j.1538-4632.2007.00708.x

[45] R Core Team. R: A Language and Environment for Statistical Computing; 2017. Available from: https://www.R-project.org/.

[46] Duong T, et al. ks: Kernel density estimation and kernel discriminant analysis for multivariate data in R. Journal of Statistical Software. 2007;21(7):1–16. doi: 10.18637/jss.v021.i07

[47] Sueur J, Aubin T, Simonis C. Seewave: a free modular tool for sound analysis and synthesis. Bioacoustics. 2008;18:213–226. doi: 10.1080/09524622.2008.9753600

[48] Organization WH. Systematic screening for active tuberculosis: principles and recommendations. World Health Organization; 2013. 25996015

[49] World Health Organization, et al. Recommendations for investigating contacts of persons with infectious tuberculosis in low-and middle-income countries. World Health Organization; 2012. 24404639

[50] Tesfaye L, Lemu YK, Tareke KG, Chaka M, Feyissa GT. Exploration of barriers and facilitators to household contact tracing of index tuberculosis cases in Anlemo district, Hadiya zone, Southern Ethiopia: Qualitative study. Plos one. 2020;15(5):e0233358. doi: 10.1371/journal.pone.0233358 32442201 PMC7244140

[51] Stresman G, Bousema T, Cook J. Malaria Hotspots: Is There Epidemiological Evidence for Fine-Scale Spatial Targeting of Interventions? Trends in parasitology. 2019;35(10):822–834. doi: 10.1016/j.pt.2019.07.013 31474558

[52] Goswami ND, Hecker EJ, Vickery C, Ahearn MA, Cox GM, Holland DP, et al. Geographic information system-based screening for TB, HIV, and syphilis (GIS-THIS): a cross-sectional study. PloS one. 2012;7(10):e46029. doi: 10.1371/journal.pone.0046029 23056227 PMC3462803

[53] Moonan PK, Oppong J, Sahbazian B, Singh KP, Sandhu R, Drewyer G, et al. What is the outcome of targeted tuberculosis screening based on universal genotyping and location? American journal of respiratory and critical care medicine. 2006;174(5):599–604. doi: 10.1164/rccm.200512-1977OC 16728707

[54] Huang CC, Trevisi L, Becerra MC, Calderón RI, Contreras CC, Jimenez J, et al. Spatial scale of tuberculosis transmission in Lima, Peru. Proceedings of the National Academy of Sciences. 2022;119(45):e2207022119. doi: 10.1073/pnas.2207022119 36322726 PMC9659349

[55] Sacchi FP, Praça RM, Tatara MB, Simonsen V, Ferrazoli L, Croda MG, et al. Prisons as reservoir for community transmission of tuberculosis, Brazil. Emerging infectious diseases. 2015;21(3):452. doi: 10.3201/eid2103.140896 25642998 PMC4344267

[56] Baussano I, Williams BG, Nunn P, Beggiato M, Fedeli U, Scano F. Tuberculosis incidence in prisons: a systematic review. PLoS Med. 2010;7(12):e1000381. doi: 10.1371/journal.pmed.1000381 21203587 PMC3006353

[57] Mabud TS, de Lourdes Delgado Alves M, Ko AI, Basu S, Walter KS, Cohen T, et al. Evaluating strategies for control of tuberculosis in prisons and prevention of spillover into communities: An observational and modeling study from Brazil. PLoS medicine. 2019;16(1):e1002737. doi: 10.1371/journal.pmed.1002737 30677013 PMC6345418

[58] Marx FM, Yaesoubi R, Menzies NA, Salomon JA, Bilinski A, Beyers N, et al. Tuberculosis control interventions targeted to previously treated people in a high-incidence setting: a modelling study. The Lancet Global Health. 2018;6(4):e426–e435. doi: 10.1016/S2214-109X(18)30022-6 29472018 PMC5849574

[59] Marx FM, Cohen T, Menzies NA, Salomon JA, Theron G, Yaesoubi R. Cost-effectiveness of post-treatment follow-up examinations and secondary prevention of tuberculosis in a high-incidence setting: a model-based analysis. The Lancet Global Health. 2020;8(9):e1223–e1233. doi: 10.1016/S2214-109X(20)30227-8 32827484 PMC7549318

[60] Sester M, Van Leth F, Bruchfeld J, Bumbacea D, Cirillo DM, Dilektasli AG, et al. Risk assessment of tuberculosis in immunocompromised patients. A TBNET study. American journal of respiratory and critical care medicine. 2014;190(10):1168–1176. doi: 10.1164/rccm.201405-0967OC 25303140

[61] Esmail H, Cobelens F, Goletti D. Transcriptional biomarkers for predicting development of tuberculosis: progress and clinical considerations. European Respiratory Journal. 2020;55(3). doi: 10.1183/13993003.01957-2019 31949119 PMC7057180

[62] Thomson DR, Rhoda DA, Tatem AJ, Castro MC. Gridded population survey sampling: a systematic scoping review of the field and strategic research agenda. International Journal of Health Geographics. 2020;19(1):1–16. doi: 10.1186/s12942-020-00230-4 32907588 PMC7488014

[63] Shaweno D, Trauer JM, Denholm JT, McBryde ES. A novel Bayesian geospatial method for estimating tuberculosis incidence reveals many missed TB cases in Ethiopia. BMC infectious diseases. 2017;17(1):1–8. doi: 10.1186/s12879-017-2759-0 28969585 PMC5625624

[64] Waller LA, Carlin BP, Xia H, Gelfand AE. Hierarchical spatio-temporal mapping of disease rates. Journal of the American Statistical association. 1997;92(438):607–617. doi: 10.1080/01621459.1997.10474012

[65] Peterson AT. Ecologic niche modeling and spatial patterns of disease transmission. Emerging infectious diseases. 2006;12(12):1822. doi: 10.3201/eid1212.060373 17326931 PMC3291346

