# Supplementary material for: Spatially-targeted tuberculosis screening has limited impact beyond household contact tracing in Lima, Peru: A model-based analysis
Source: PLoS One. 2023 Oct 30;18(10):e0293519. doi: 10.1371/journal.pone.0293519 (PMC10615320; doi:10.1371/journal.pone.0293519)
Supplement: S1 Appendix — Supporting information includes household size distribution, further details on transmission model states and parameters, further details on calibration methods and results, resampled parameter value distributions, alternative calibration methods and intervention result based on different sensitivity analyses. (PDF) [file pone.0293519.s001.pdf]

Supplement: Spatially-targeted tuberculosis screening has limited impact beyond household contact tracing in Lima, Peru: a model-based analysis

S1 Distribution of at-risk individuals and households

We used unadjusted *Worldpop* data for Peru from 2009 [1] to obtain estimates of local population density within SMP. *Worldpop* divides areas into $\approx 100\ m^2$ grid cells and provides estimated counts for the total number of people in each cell. In SMP, there were an estimated total of $\approx 615,800$ people with an average of ≈ 140 individuals per grid cell. See Figure 1 for the estimated population density of SMP.

To initialize the model, we obtained geographic coordinates of SMP using the GADM database of global administrative areas [2]. We then divided our spatial area into grid cells to match the *Worldpop* data. For each grid cell, we sampled households with membership sizes drawn from the empirical distribution of household sizes in Peru in 2007 obtained from United Nations data [3]. The United Nations dataset has counts by household size i.e., total number of households with 1 person, 2 people and so on with all households of size ≥ 6 grouped into a single count. To recreate the empirical distribution, we sampled households of size 1 to 6 with the probability of sampling a 6 person household equaling the total number of households ≥ 6 divided by the total number of households. To create a realistic distribution of larger household sizes, for each 6 sampled, we resampled the household size from a triangular distribution with minimum household size of 6, maximum of 9 and a mode of 6. Doing this ensured that there were fewer households with more people (e.g., there were more 6 person households than 7 person households). We chose a maximum of 9 people because this best recreated the empirical distribution. Finally, for a given grid cell, we sampled households until total number of people was greater than or equal to the corresponding grid cell in the *Worldpop* data. If the total number of people was greater than the *Worldpop* data, we removed the last house from the grid cell. This process resulted in a slightly lower population than the *Worldpop* data. Within a grid cell, we assumed all households were located near the centroid since we did not have coordinates for households in SMP. Below are the distribution of household sizes in an example simulated population and in the empirical distribution from 2007 in Peru [3]. We created 10 realizations of the population of SMP which we then used to simulate disease transmission.

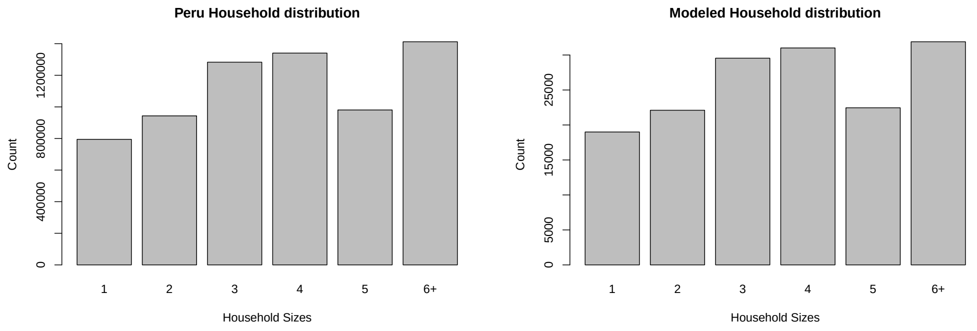

(a) 2007 Peru Household Distribution (b) Simulated Population Household Distribution

Figure S1: Distribution of household sizes in the data (left) and model (right)

S2 Transmission parameter derivations

To derive our transmission rates, we first sampled unscaled values for the household transmission rate, β_{uHH}, and calculated the following:

$$\beta_{HH} = \frac{\beta_{uHH}}{Pop_{HH} - 1}$$

where β_{uHH} is the sampled unscaled household transmission rate, and Pop_{HH} is the average number of individuals per household across the entire modeled district.

Next, to derive the community transmission rate, β_C, we scaled by average the number of community contacts per individual, but also accounted for the fact that the probability of transmission attenuates by distance:

$$\beta_C = \beta_{scalar} \frac{\beta_{uHH}}{mean(Dist_{power}) Pop_{Centroid}}$$

where β_{scalar} is a sampled reduction factor such that the per contact $\beta_C < \beta_{HH}$. $Dist_{power}$ sums all the distances from a given centroid to all other centroids (within the spatial range, θ) and transforms them by the power kernel, i.e., for the distance between centroids 'a' and 'b': $d_{ab}^{-\alpha}$. This is multiplied by $Pop_{Centroid}$ or the average number of people per centroid. See Table 1 for all sampled parameter values.

S3 Transmission Model States and Parameters

Individuals are born susceptible (S). Susceptible as well as latent and recovered individuals may become infected according to the force of infection. Once infected, individuals transition to the early latent state (EL) from which ≈ 5 to $\approx 10\%$ of individuals progress to infectious active TB (I) within 5 years of infection (rates of progression decrease with each year since infection). The remaining individuals transition to the late latent state (LL) from which only ≈ 5 to $\approx 10\%$ of individuals transition to I over the course of their lifetime. Finally, individuals in I may recover either spontaneously or through treatment, and transition to the R compartment. Individuals may die from natural causes in any state and are immediately replaced with a susceptible individuals. Furthermore, those in I may also die from TB. Upon death from TB, individuals may be replaced with either a susceptible, latent or recovered individual. The probability of each of these replacements is based on the relative distribution of disease states in the population. For instance, if there are more latent individuals, someone who dies from TB is more likely to be replaced with another latent individual. We implemented this scheme to avoid always placing a susceptible individual in an area with high transmission as this would cause the pool of susceptible individuals to be artificially replenished and overestimate the amount of transmission that occurs in these areas.

In addition to the disease states, we also modeled an intervention state such that detected individuals with active TB are given treatment (T), assumed to no longer be infectious, eventually recover and move to R. See Table 1 for all parameter values and ranges used in the model and Figure 2 for the model schematic.

S4 Calculation of Active TB Treatment Rate

To derive the active TB treatment rate, we sampled values for the case detection rate (80% ±10%) using a range obtained from the estimate for Peru over the course of the study [4]. We next accounted for competing risks using the following equation.

$$ATB_{treat} = (CDR\mu + CDR\kappa + CDR\gamma)/(1 - CDR) \tag{1}$$

where CDR is the case detection rate, μ is the mortality rate, κ is the active TB mortality rate and γ is the spontaneous recovery rate among those with active TB. We assumed that all detected individuals were immediately given treatment.

S5 Calibration Methods: Recreating Local Intensity of Cases

To calibrate our model to the spatial distribution of the observed data, we used sample-importance-resampling [5]. See Figure S2 for an overview of the main calibration methods.

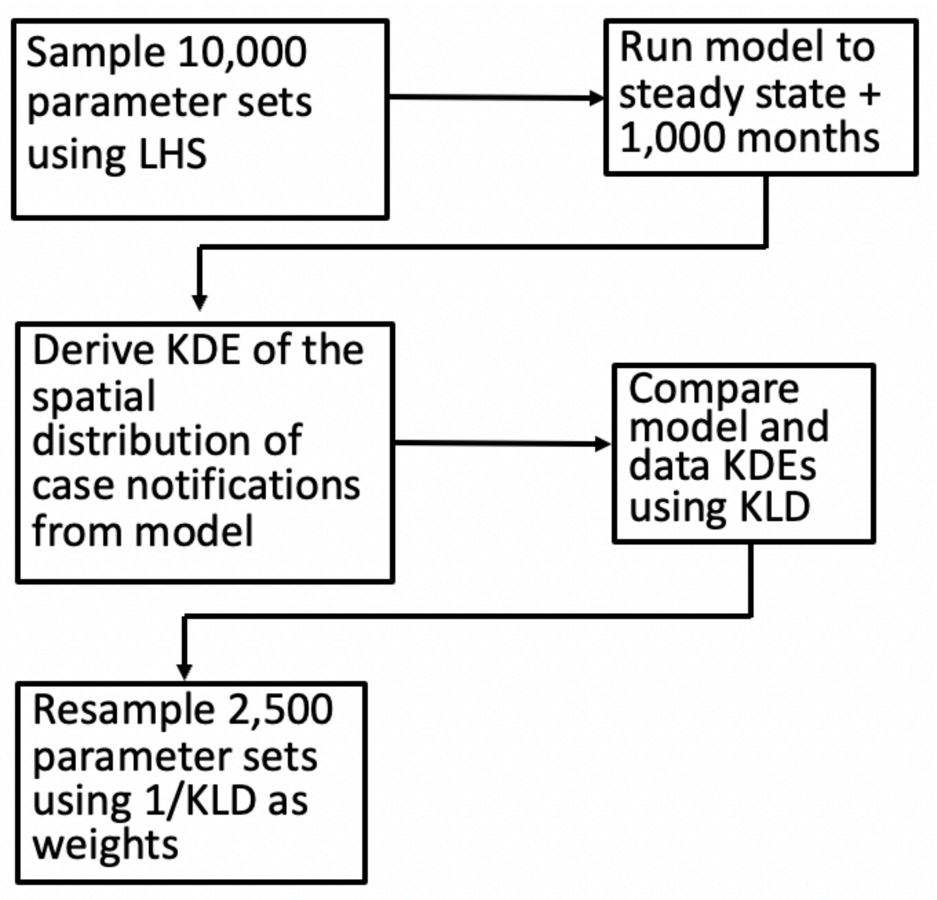

Figure S2: Schematic of the main calibration methods: (1) In the 'Sample' step, we sample 10,000 parameter sets using latin hypercube sampling (LHS), (2) the model is then run to steady state plus an additional 1,000 one-month time steps post-steady state, (3) In the 'Importance' step, kernel density estimates (KDE) are derived from the model for each 33-month slice of the 1,000 post-steady state time steps (e.g., month 1-33, 2-34 etc.) and the model KDE and data KDE are compared using the Kullback-Leibler divergence (KLD), (4) finally, in the 'Resampling' step, the parameter sets are are resampled with replacement 2,500 times using 1/KLD as weights.

Sample To account for uncertainty in parameter values, we ran the model with 10,000 parameter sets obtained using latin hypercube sampling [6]. For each parameter set, a population distribution realization of SMP was randomly selected. We then seeded the model with infectious individuals in the area with the highest intensity of cases observed in the study data. To account for uncertainty in seeding, we varied the radius within which infectious seeds were dispersed. The model was run to steady state and then for an additional 1,000 one-month times steps which were used for calibration.

Importance To determine how well a given parameter set recreated the local case intensity study data, we examined all possible 33-month slices (i.e., this represents the simulated study period and is the length of time that SMP was in the study.) of the model output from the 1,000 one-month time steps post-steady state. We systematically explored the model output in this way, because once at steady state, the spatial distribution of cases may continue to change. We did not have information about the age of the transmission cluster, therefore we assumed that data from our study was generated at steady-state.

We first excluded all 33-month slices in which (1) the number of case notifications (during this time period) were outside the target range of 799 $\pm 25\%$ cases, (2) the mean latent TB prevalence that would likely be detected by the tuberculin skin test (i.e., EL, LL and R) was outside the target range of $\approx 20\%$ to $\approx 30\%$ [7] or (3) the approximate proportion of detected active TB cases was outside the target range of 80% to 90%.

Next, among included 33-month slices, we calculated kernel density estimates (KDEs) of the 2-dimensional (i.e., latitude and longitude) spatial distribution of cases from the model output [8]. We then measured the difference between each model KDE to a KDE of the case distribution from the data using the Kullback-Leibler divergence (KLD) [9]. Finally, for each parameter set, we selected the lowest KLD which corresponds to the best-calibrating 33-month slice. We used this value for resampling i.e., each parameter set was associated with a single KLD derived from a single 33-month time period. We also examined other methods of calibration e.g., $\mid I_{data} - I_{model} \mid$ by centroid or by groups of centroids (based on neighbor).

Resampling We resampled 2,500 times from all parameter sets with replacement using the inverse of the KLD as weights. Overall, we obtained an array of parameter sets that most closely recreate the study data.

In addition to using Kullback Leibler Divergence (KLD) to compare the model and data kernel density estimates from each parameter set, we explored other potential methods of calibration. Specifically, we compared model outputs to the data by groups of centroids. We wanted to ensure that similar case intensities in the same regions (but not necessarily the same centroid) performed better than similar case intensities in distal regions. Therefore, for each centroid, we obtained all adjacent neighbors (using a 'queen' definition), and then took all neighbors of the adjacent neighbors and repeated until 5th degree neighbors were obtained. We then calculated $\mid I_{data} - I_{model} \mid$ for each group and summed over the entire district. As a sensitivity analysis we did the same for 3rd degree neighbor groups, but could not form groups higher than 5th degree neighbors due to limitations in computational power. See Figure S10 for more details.

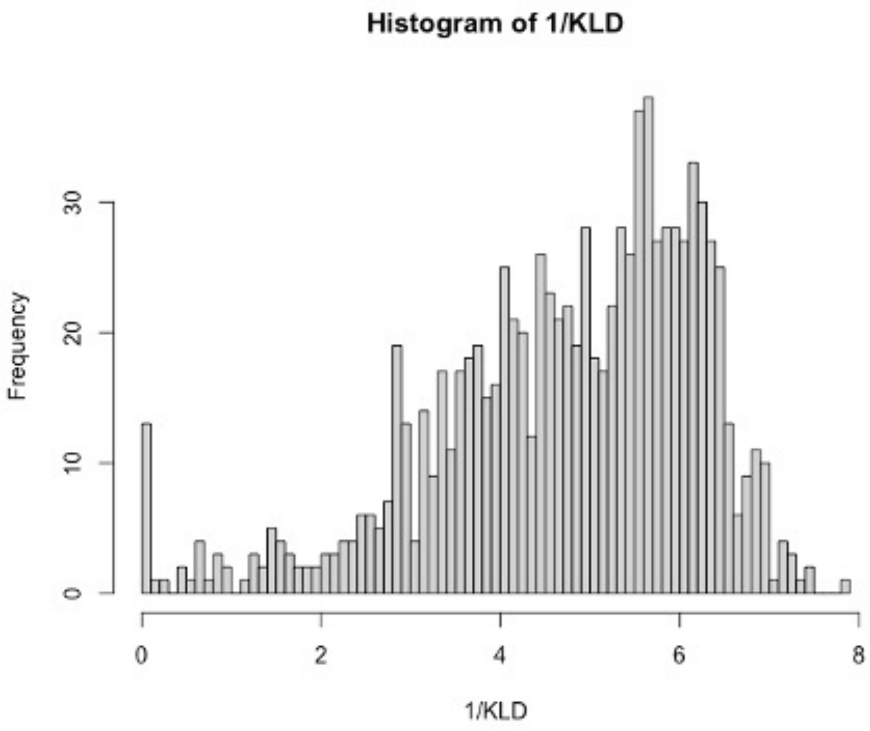

Figure S3: Distributions of sampling weights. In total 839 of the 10,000 initial parameter sets were sampled.

S6 Calibration results

Comparisons between the best-calibrating parameter set and data are shown in Figure S4. Other calibration metrics including the proportion of co-prevalent household cases (Figure S5), the relationship between latent TB prevalence and number of detected and undected active TB cases (Figure S6), the approximate proportion of detected TB cases (Figure S7), and distributions of resampled parameters values are shown as well (Figure S9).

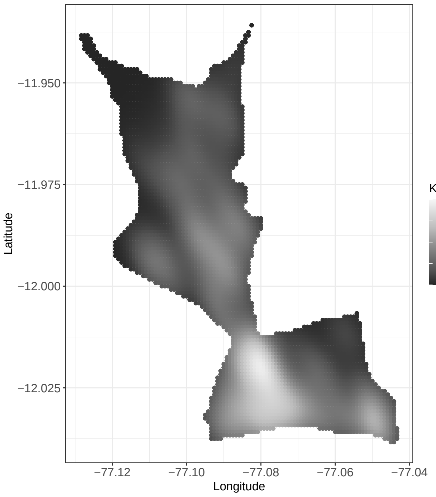

(a) Data Kernel Density Estimate

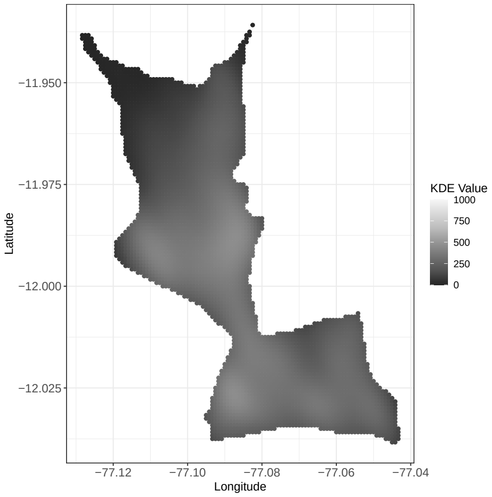

(b) Best-Calibrating Run from Kullback Leibler Method Kernel Density Estimate

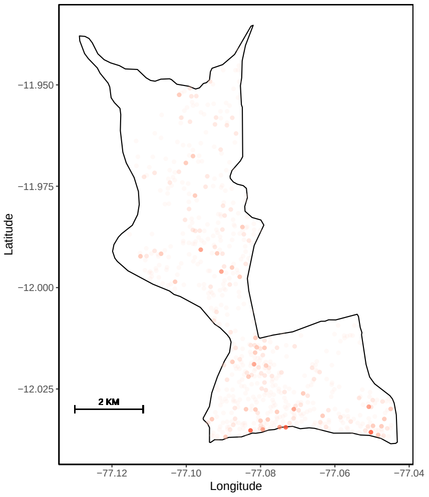

(c) Data Distribution of Cases

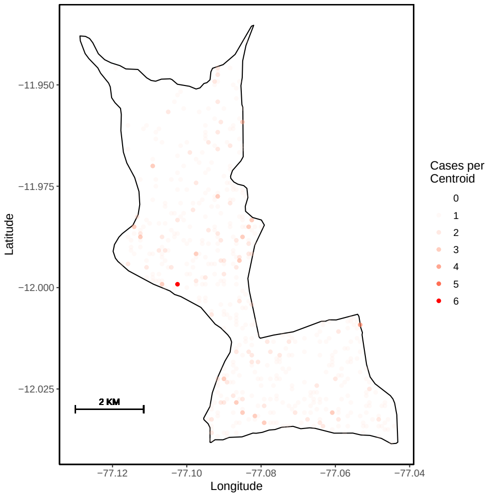

(d) Best-Calibrating Parameter Set Distribution of Cases

Figure S4: (a) Kernel Density Estimate from the cohort Study (b) Kernel Density Estimate from the best calibrating parameter set (c) Jittered distribution of cases from the cohort study (d) Distribution of cases from the best calibrating parameter set. The data reflects all case notifications during the 33-month cohort study and the best-calibrating parameter set output reflects all case notifications from the 33-month simulated study period.

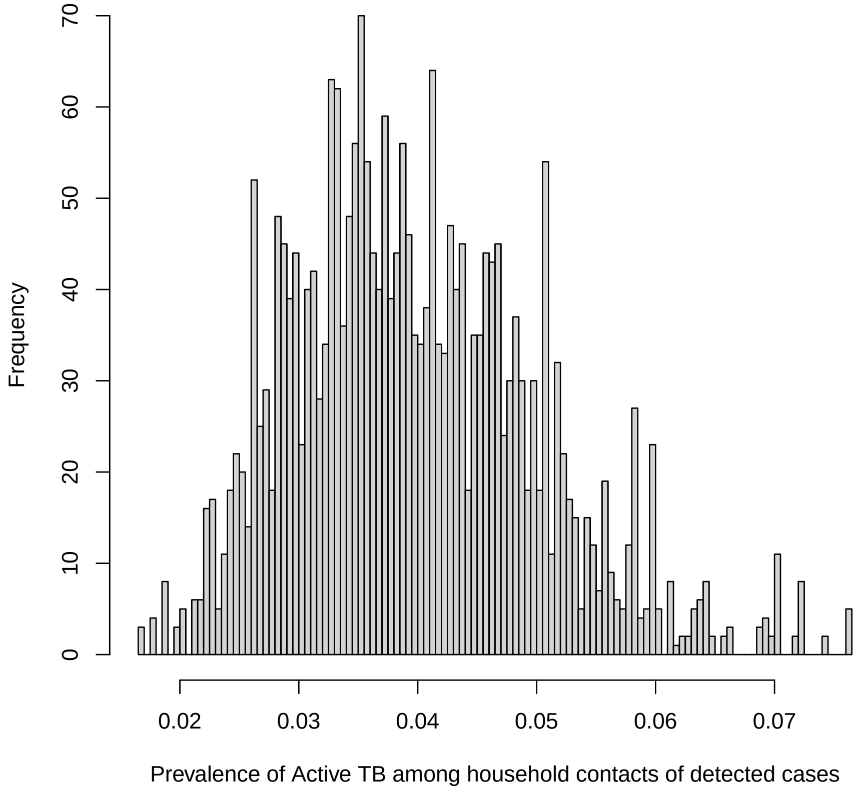

Figure S5: Proportion of household contacts with active TB during the simulated study period (i.e., the 33 month slice that is calibrated to the data). For each newly detected index case, we added the number of household contacts that either have active TB or are currently undergoing treatment and divide by the total number of household contacts.

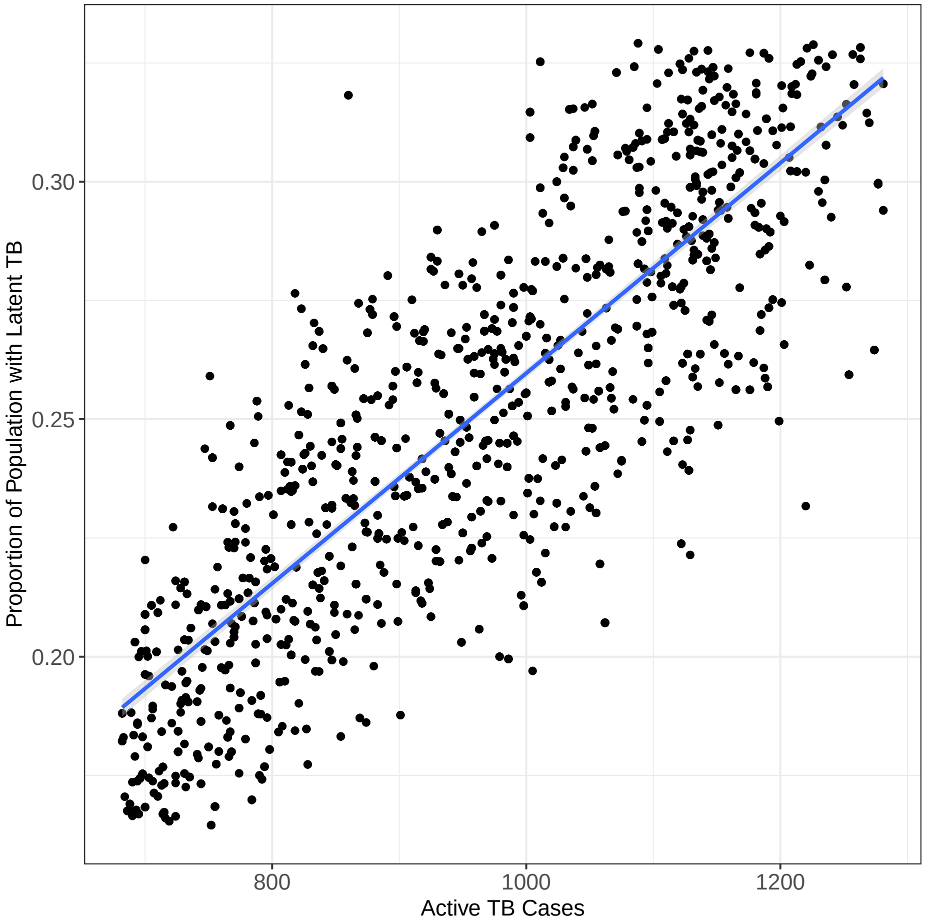

Figure S6: Mean proportion of individuals with latent TB among resampled parameter sets over the course of the study by total number of incident cases (i.e., detected and undetected).

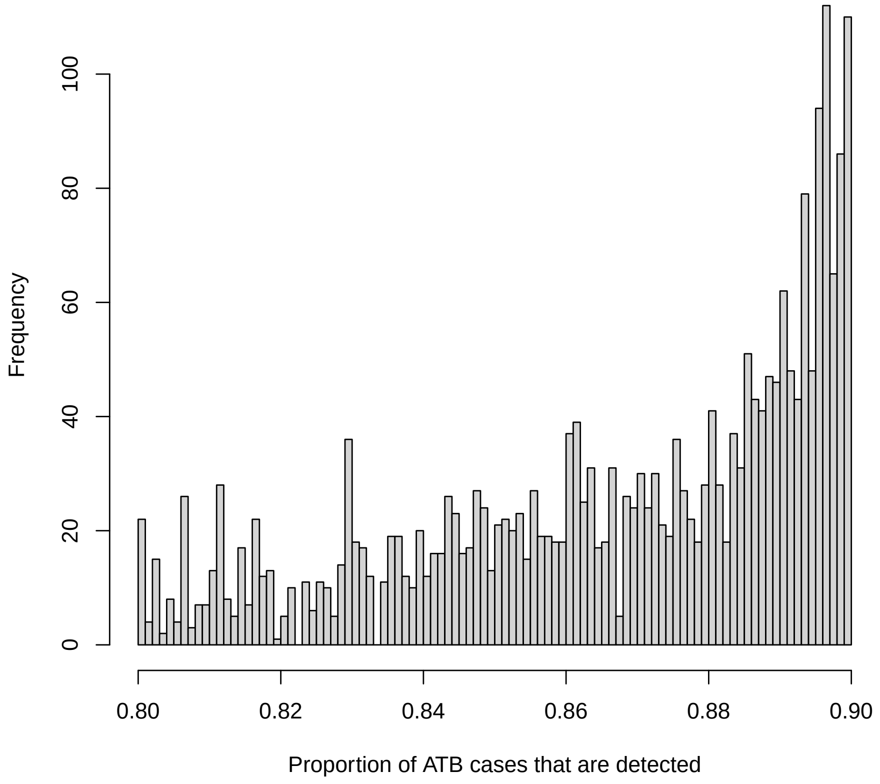

Figure S7: Approximate proportion of active TB cases among resampled parameter sets that are detected. To calculate, we divided the number of individuals that transitioned to treatment by the number of incident cases (i.e., individuals that transitioned to active TB) that occurred over the course of the study.

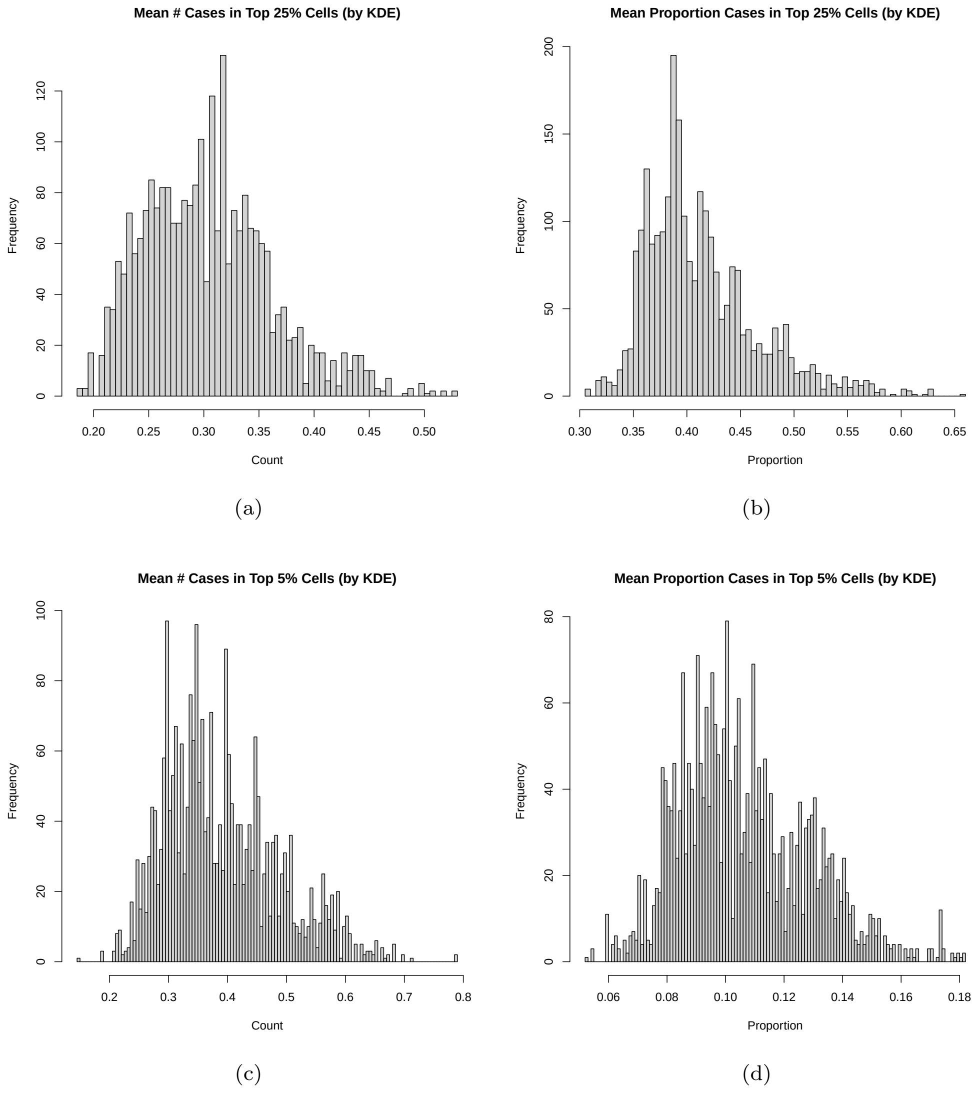

Figure S8: High prevalence grid cell metrics: (a) average number of cases per grid cell among top 25th percentile cells according to kernel density estimate, (b) average proportion of cases among top 25th percentile grid cells according to kernel density estimate (c) average number of cases per grid cell among top 5th percentile cells according to kernel density estimate, (d) average proportion of cases among top 5th percentile grid cells according to kernel density estimate

The proportion of co-prevalent household cases of active TB was consistent with the literature [10]. Additionally, the prevalence of latent TB corresponded to ≈ 20 to ≈ 30% of the population which is consistent with what has been estimated for Peru [7]. We assumed that the proportion of detected active TB cases was between 80% and 90%. Finally, the numbers of cases per grid cell are low with an average number of cases in the top 5% and top 25% grid cells (i.e., those with the higher kernel density estimates) being below 1.

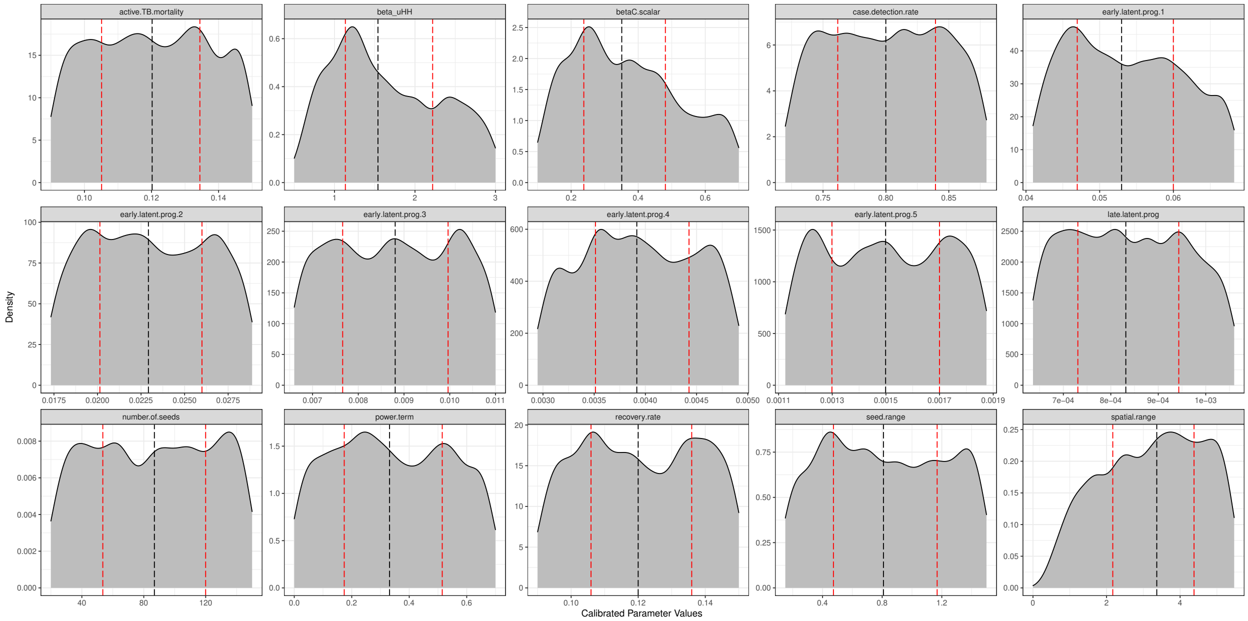

Figure S9: Distributions of resampled parameter values sets with 25th and 75th percentiles in red and the median value in black. Where 'active TB morality' is the mortality rate among individuals with active TB, '$\beta_u HH$' is the unscaled household transmission rate, '$\beta_c Scalar$' is the scaler multiplied by the household transmission rate to derive the community transmission rate, 'case detection rate' is the rate at which individuals with active TB are detected, 'early latent prog' are the early latent rates of progression to active TB number by year since infection, 'late latent prog' is the late latent rates of progression to active TB, 'number of seeds' is the number of infectious seeds at the start of the simulation, 'power term' is the power term used in the power transmission kernel, 'recovery rate' is the rate of spontaneous recovery for individuals with active TB, 'seed range' is the area within which infectious individuals are seeded, and 'spatial range' is the spatial range of community contacts. See Table 1 for details on parameters and their initial ranges.

S7 Alternative Calibration Methods

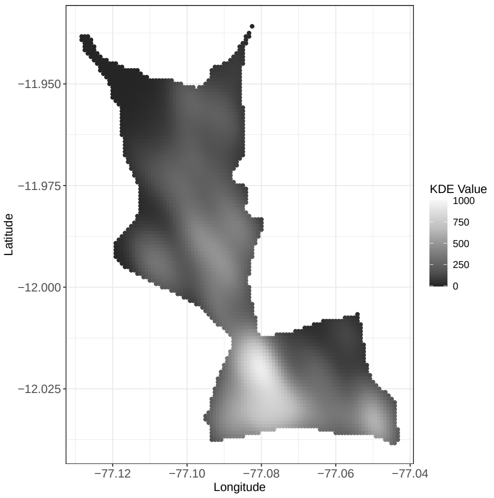

(a) Data

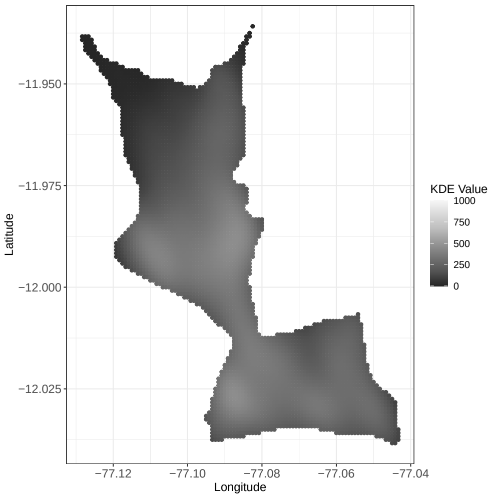

(b) Best-Calibrating Run from Kullback Leibler Method

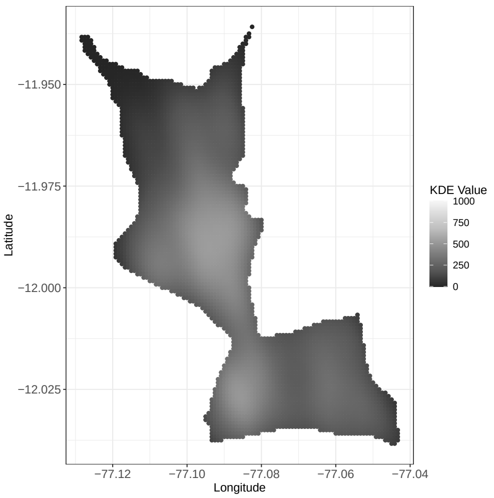

(c) Best-Calibrating Run from 5th degree neighbors method

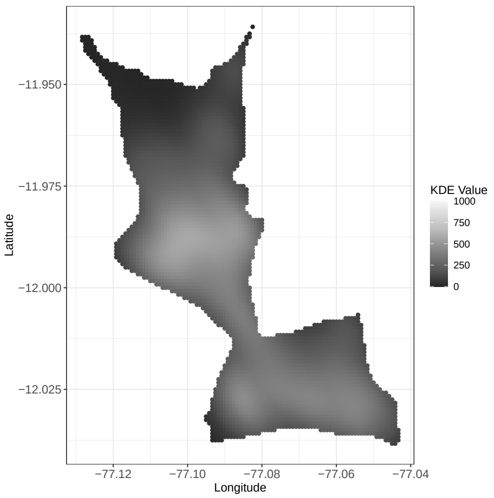

(d) Best-Calibrating Run from 3rd degree neighbors method

Figure S10: (a) Kernel Density Estimate from Lima Study (b) Kernel Density Estimate from best fitting Kullback Leibler Divergence selected parameter set (c) Kernel Density Estimate from best-fitting 5th degree neighbors selected parameter set (d) Kernel Density Estimate from best-fitting 3rd degree neighbors selected parameter set. The data reflects all case notifications during the 33-month cohort study and the best-calibrating parameter set output reflects all case notifications from the 33-month simulated study period.

Overall, the data appears to have a higher intensity of cases (i.e., high transmission areas) in the bottom left of the district. Because the graphical fit does not appear to be a perfect match, the choice between methods is not obvious. Therefore, we examined how well the model fits the global spatial structure of the data and ran the interventions on resampled parameter sets from the 5th degree neighbors method as a sensitivity analysis.

S8 Calibration Methods: Recreating the Global Spatial Structure of the Data

To further confirm our calibration methods, we calculated two global spatial metrics among resampled parameter sets assessing the match between the model and the global spatial structure of the data.

First, we examined whether the selected parameter sets perform better than randomly shuffling centroids throughout the district through a permutation test. For all resampled parameter sets, we calculated the 5th degree neighbors metric. This metric is described as an alternative calibration method in main text, but we are using this metric as part of the permutation test to account for spatial proximity. Then, we shuffled the centroids (i.e., the distribution of cases) in the model output and recalculated this metric. We repeated this 10,000 times and then compared the 5th degree neighbors metric of the model output to all permutations i.e., we determined what percentile the model output value is within the 10,000 permutations (see Figure S11). Lower ranked parameter sets (x-axis) correspond to those that calibrated to the data better and lower percentiles corresponded to the model performing better than the majority of random permutations.

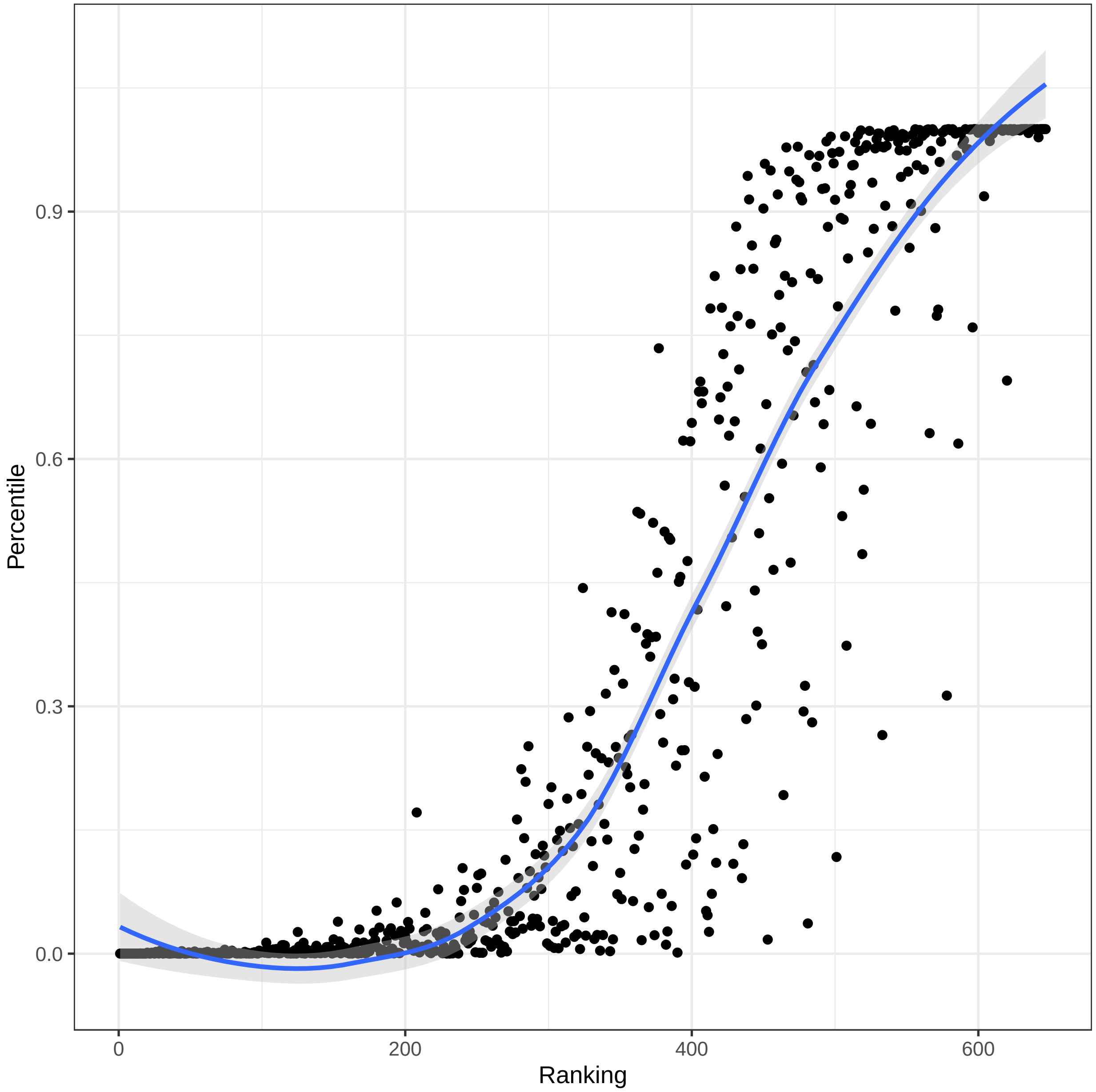

Figure S11: Results from the permutation test comparing the performance of the model to 10,000 random permutations of the distribution of cases. The x-axis displays the parameter set rankings according to the KLD calibration method (i.e., lower rankings correspond to parameter sets that calibrate to the data better). The y-axis displays where the model output falls in the distribution of random permutations. For instance, 0.75 means that 75% of random permutations perform better than the model output. The blue line is a spline calculated using the LOESS method in R [11] and the shaded region represents 95% confidence intervals.

As expected, parameter sets with lower KLD values (i.e., those that calibrated to the data better) performed better than the majority of random permutations of the distribution of cases.

Next, to examine how well our model matches the global spatial autocorrelation in the data, we calculated global Moran's I [12, 13]. We derived a unique spatial weights matrix (for the data and model output) for each parameter set by incorporating the spatial range and transforming distances between centroids by α the power parameter. In other words, the spatial weights matrix reflected how centroids were connected via the power transmission kernel. We calculated Moran's I using the MCMC permutation test command 'moran.mc' in the R package 'spdep' [14] and compared the data to the model across the entire distribution of resampled parameter sets S12.

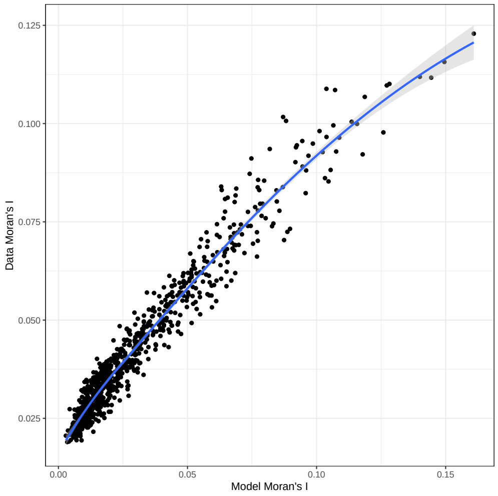

Figure S12: Correspondance between data and model derived Moran's I across all resampled parameter sets.

Spatial autocorrelation of simulations from resampled parameter sets matched the data well with an R^2 value of 0.95.

S9 Sensitivity Analysis: Interventions run on 5,000 resampled Parameter Sets

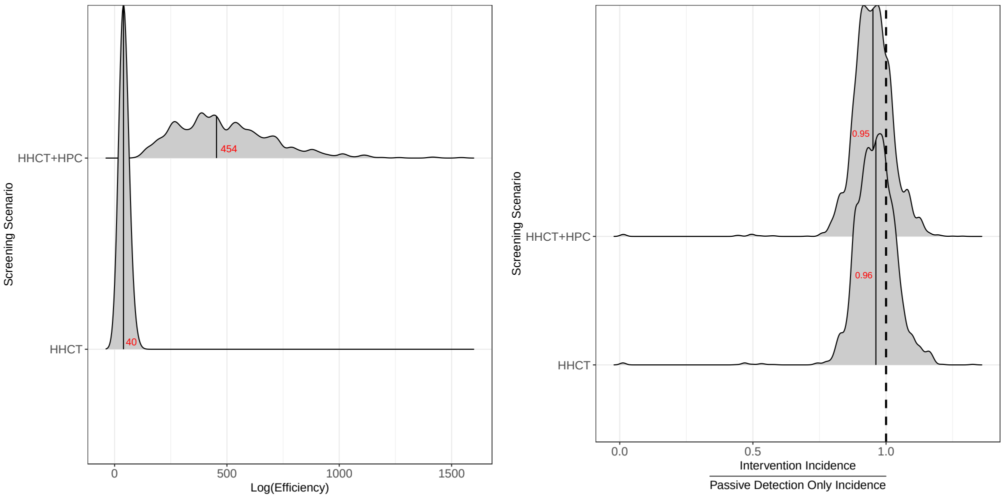

(a) Efficiency of Interventions (b) Protective Impacts of Interventions

Figure S13: Ridgeline plots displaying (a) The efficiency of different screening interventions. 'Efficiency' was defined as the number of individuals that need to be screened to find a case of active TB and (b) Rate ratios comparing the 5-year incidence rate of different screening interventions to passive surveillance only. Medians are in red.

S10 Sensitivity Analysis: Interventions run on 5th degree neighbors Calibrated Parameter Sets

We ran all interventions on resampled parameter sets using the 5th degree neighbors calibration method. Conclusions from this analysis were consistent with our main analysis which used the KDE and KLD method of calibration.

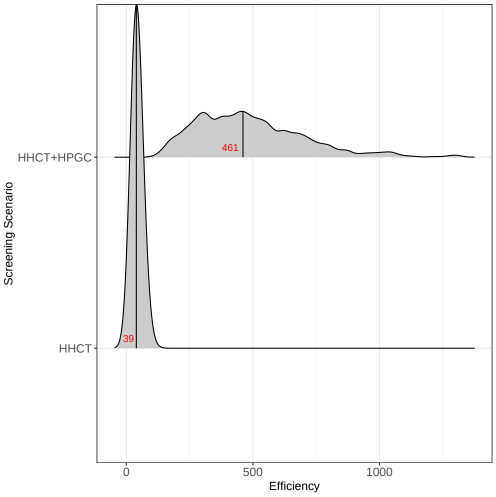

(a) Efficiency of Interventions

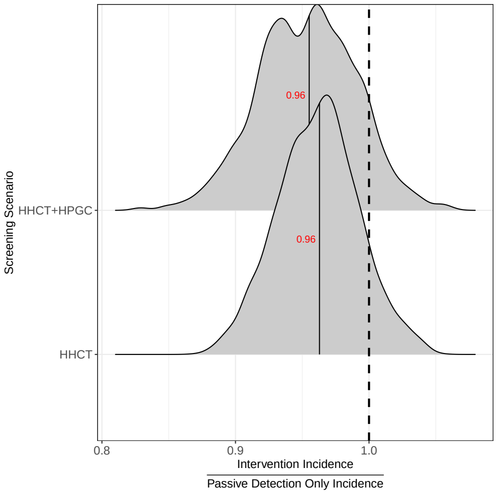

(b) Protective Impacts of Interventions

Figure S14: Ridgeline plots displaying (a) The efficiency of different screening interventions. 'Efficiency' was defined as the number of individuals that need to be screened to find a case of active TB and (b) Rate ratios comparing the 5-year incidence rate of different screening interventions to passive surveillance only. Medians are in red.

S11 Sensitivity Analysis: Performance of Interventions Varying Intensity of Transmission Over Space

We examined the sensitivity of our results to changes in key parameter values affecting the intensity of transmission over space.

First, with respect to efficiency or the number of individuals that need to be screened to find a case of active TB, we see emergent trends in that the efficiency of interventions increase with increases household transmission rate values and decrease with increasing community transmission rates (Figure S15).

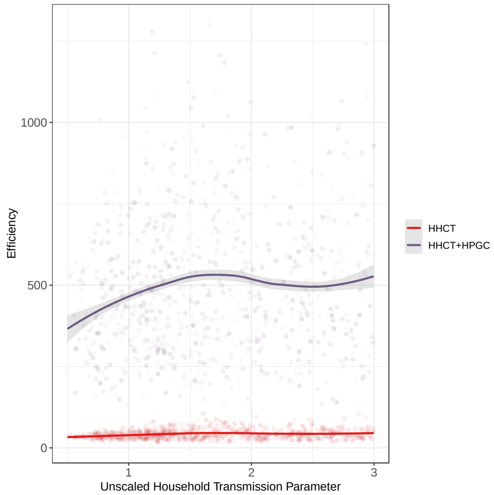

(a) Efficiency of Interventions by β_{HH}

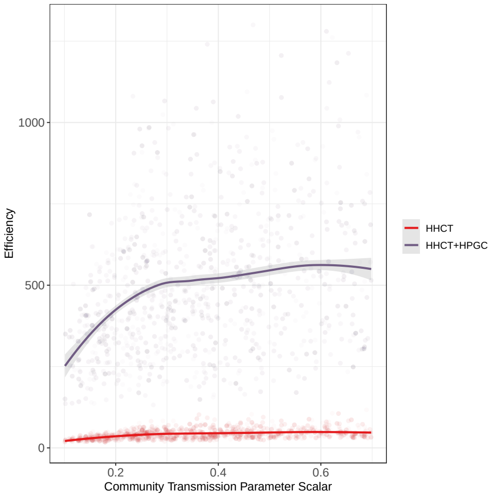

(b) Efficiency of Interventions by β_C

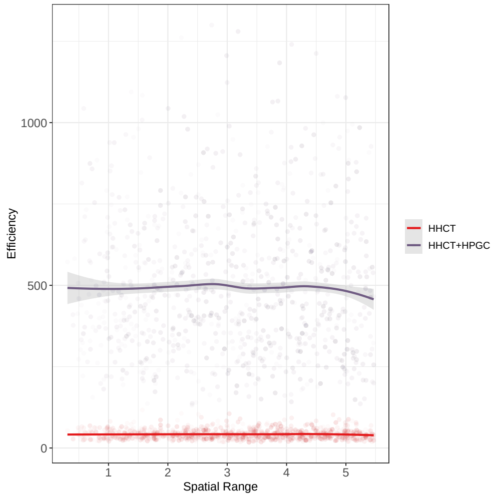

(c) Efficiency of Interventions by Spatial Range (θ)

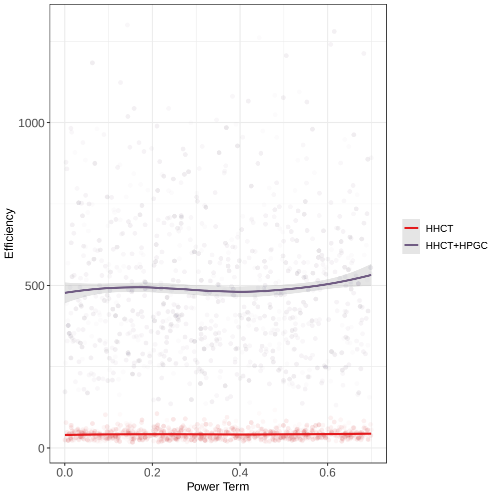

(d) Efficiency of Interventions by Power Term (α)

Figure S15: (a) Efficiency of different screening interventions by household transmission rate. (b) Efficiency of different screening interventions by community transmission rate. (c) Efficiency of different screening interventions by spatial range restricting community transmission. (d) Efficiency of different screening interventions by community transmission kernel power term. For all plots, lines are splines calculated using the LOESS method in R [11]. Shaded regions represent 95% confidence intervals.

Next, with respect to the impacts of interventions or the reduction the 5-year incidence rate, we do not see any emergent trends (Figure S16).

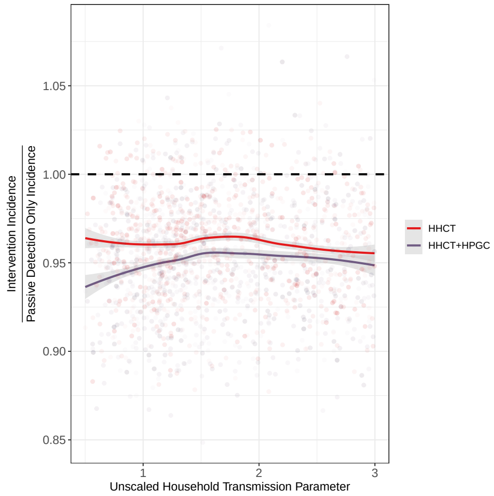

(a) Protective Impacts of Interventions by β_{HH}

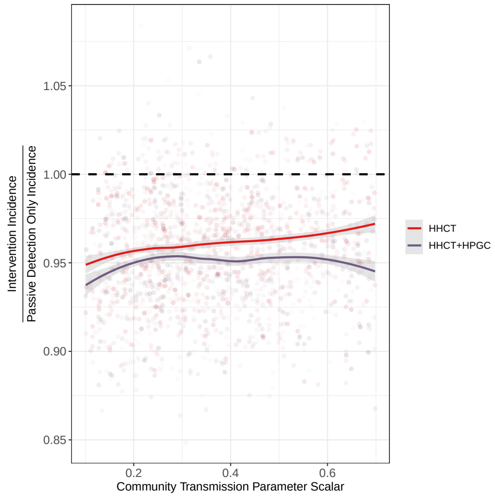

(b) Protective Impacts of Interventions by β_C

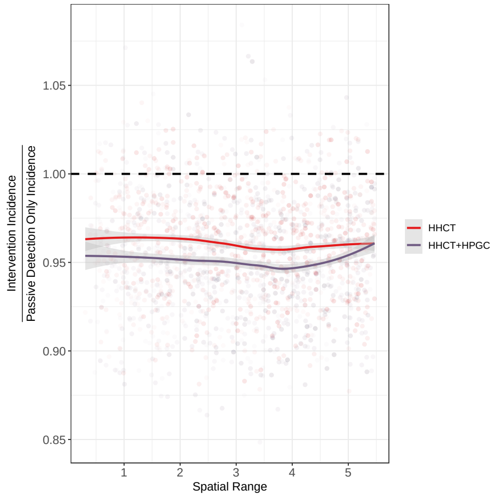

(c) Protective Impacts of Interventions by Spatial Range (θ)

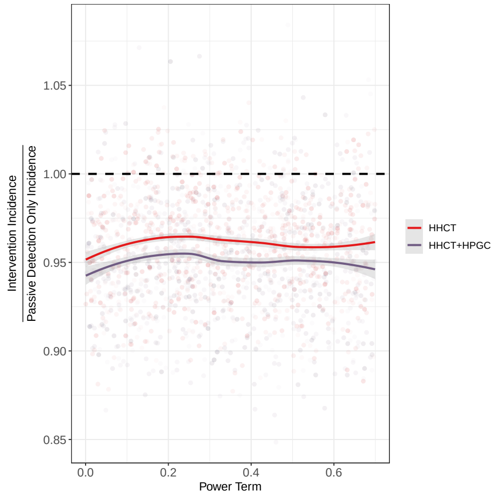

(d) Protective Impacts of Interventions by Power Term (α)

Figure S16: (a) Rate ratios of different screening interventions by household transmission rate. (b) Rate ratios of different screening interventions by community transmission rate. (c) Rate ratios of different screening interventions by spatial range restricting community transmission. (d) Rate ratios of different screening interventions by community transmission kernel power term. For all plots, lines are splines calculated using the LOESS method in R [11]. Shaded regions represent 95% confidence intervals.

S12 Sensitivity Analysis: Performance of Interventions using 70% Diagnostic Sensitivity

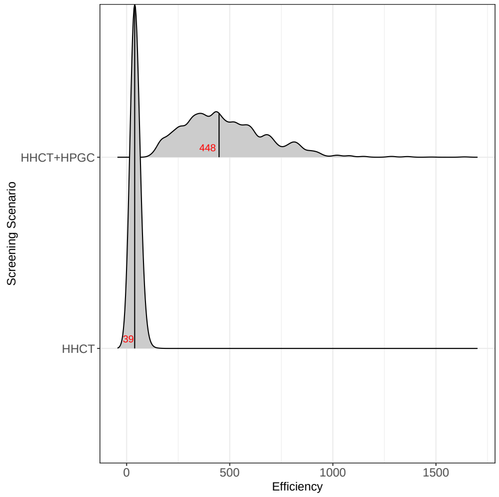

(a) Efficiency of Interventions

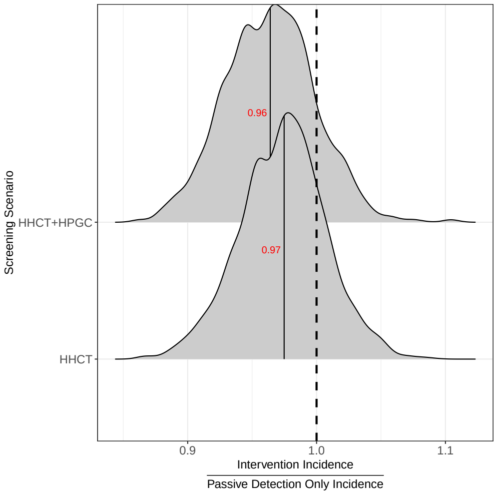

(b) Protective Impacts of Interventions

Figure S17: Ridgeline plots displaying (a) The efficiency of screening interventions with 70% diagnostic sensitivity. 'Efficiency' was defined as the number of individuals that need to be screened to find a case of active TB and (b) Rate ratios comparing the 5-year incidence rate of screening interventions with 70% diagnostic sensitivity to passive surveillance only. Medians are in red.

S13 Sensitivity Analysis: Performance of Interventions Using Case Notifications as Weights for HPGC

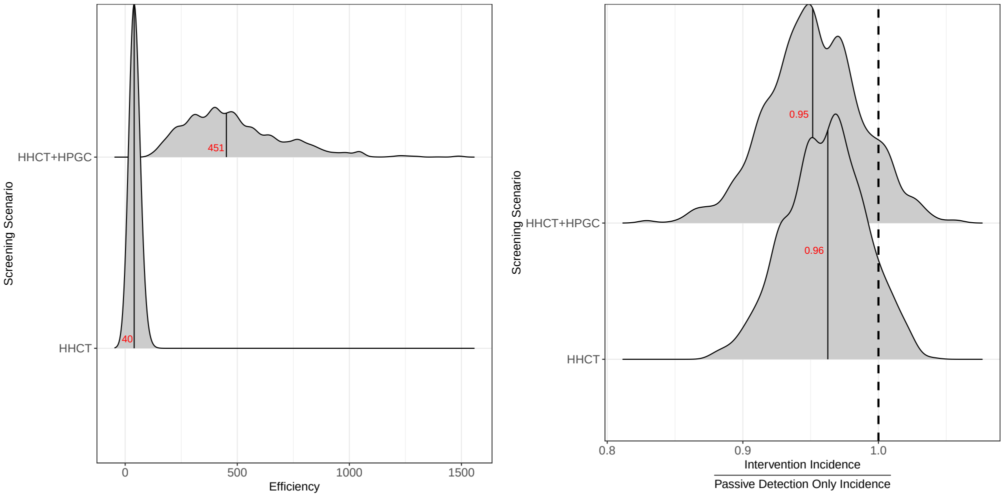

(a) Efficiency of Interventions (b) Protective Impacts of Interventions

Figure S18: Ridgeline plots displaying (a) The efficiency of screening interventions using case notifications as weights. 'Efficiency' was defined as the number of individuals that need to be screened to find a case of active TB and (b) Rate ratios comparing the 5-year incidence rate of screening interventions using case notifications as weights to passive surveillance only. Medians are in red.

S14 Sensitivity Analysis: Performance of Interventions With Scaled Up Screening Area

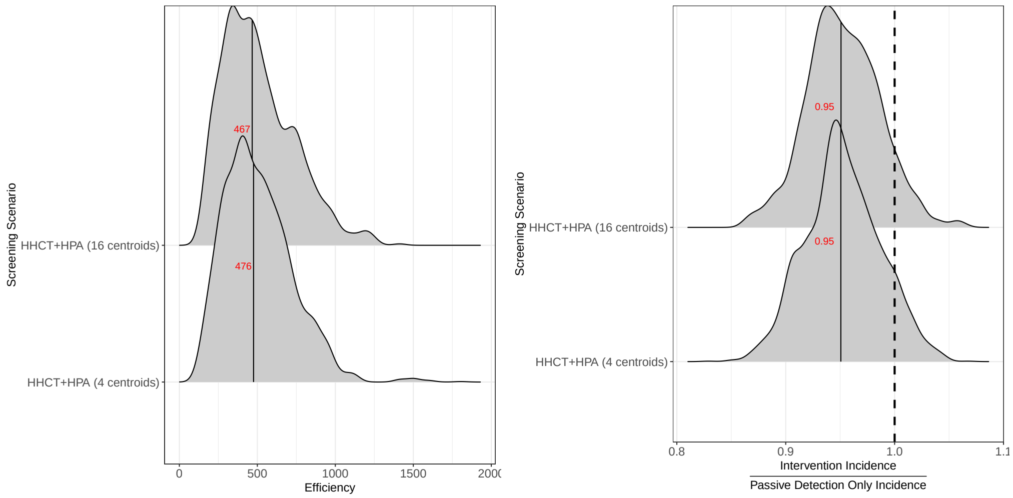

(a) Efficiency of Interventions

(b) Protective Impacts of Interventions

Figure S19: Ridgeline plots displaying (a) The efficiency of screening interventions with different screening areas (HPA is high prevalence area). 'Efficiency' was defined as the number of individuals that need to be screened to find a case of active TB and (b) Rate ratios comparing the 5-year incidence rate of screening interventions to passive surveillance only. Medians are in red.

S15 Sensitivity Analysis: Performance of Interventions With Scaled Up Screening Area and Coverage

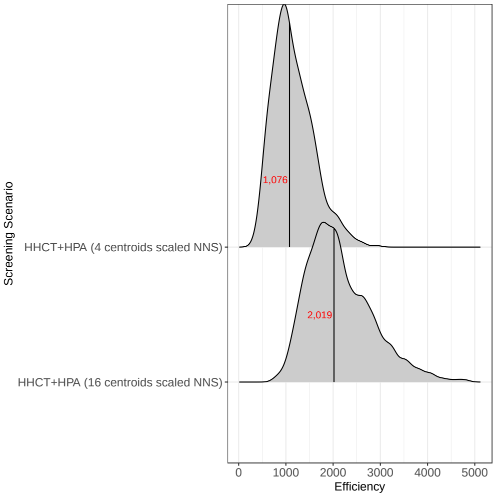

(a) Efficiency of Interventions

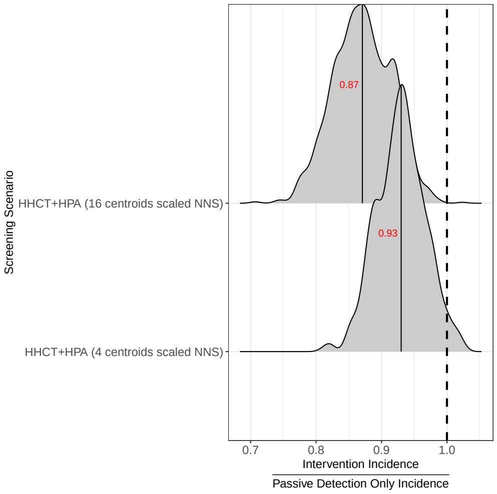

(b) Protective Impacts of Interventions

Figure S20: Ridgeline plots displaying (a) The efficiency of screening interventions with different screening areas and coverage (HPA is high prevalence area). 'Efficiency' was defined as the number of individuals that need to be screened to find a case of active TB and (b) Rate ratios comparing the 5-year incidence rate of screening interventions to passive surveillance only. Medians are in red.

S16 Performance of interventions over 10 years

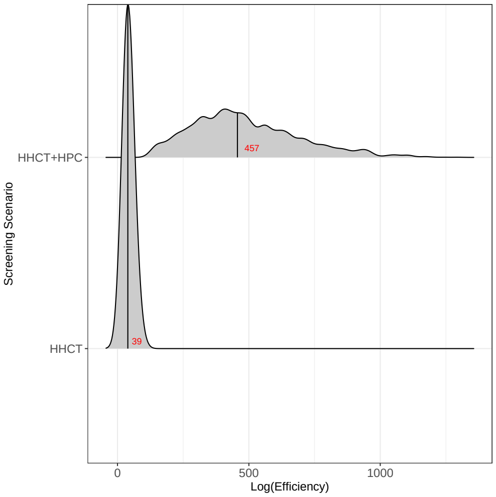

(a) Efficiency of Interventions

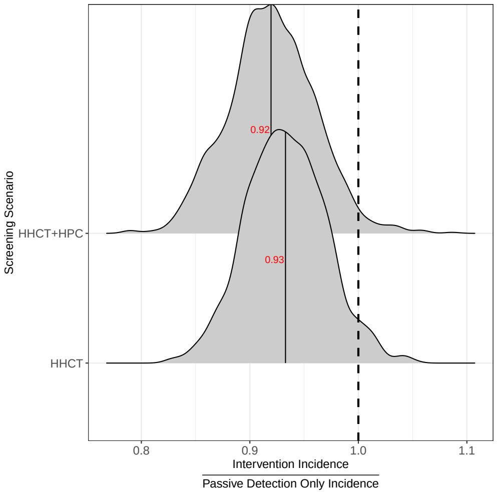

(b) Protective Impacts of Interventions

Figure S21: Ridgeline plots displaying (a) The efficiency of screening interventions over 10 years. 'Efficiency' was defined as the number of individuals that need to be screened to find a case of active TB and (b) Rate ratios comparing the 10-year incidence rate of screening interventions to passive surveillance only. Medians are in red.

References

[1] (www worldpop org School of Geography W, Environmental Science UoSDoG, Geosciences UdN University of Louisville; Departement de Geographie, Center for International Earth Science Information Network (CIESIN) CUGHRPDPFbTB, (OPP1134076) MGF. The spatial distribution of population in 2009, Peru; 2018. Available from: https://dx.doi.org/10.5258/SOTON/WP00645.

[2] Areas GA. GADM database of global administrative areas. Global Administrative Areas. 2012.

[3] Peru 2007 Households by age and sex of reference person and by size of household.;. Accessed: 2020-06-09. http://data.un.org/Data.aspx?d=POP&f=tableCode:50;.

[4] WHO TB burden estimates;. Accessed: 2021-05-17. https://www.who.int/teams/global-tuberculosis-programme/data.

[5] Rubin DB. Using the SIR algorithm to simulate posterior distributions. Bayesian statistics. 1988;3:395-402.

[6] Stein M. Large sample properties of simulations using Latin hypercube sampling. Technometrics. 1987;29(2):143-51.

[7] Houben RM, Dodd PJ. The global burden of latent tuberculosis infection: a re-estimation using mathematical modelling. PLoS medicine. 2016;13(10):e1002152.

[8] Deng H, Wickham H. Density estimation in R. Electronic publication. 2011.

[9] Kullback S, Leibler RA. On information and sufficiency. The annals of mathematical statistics. 1951;22(1):79-86.

[10] Fox GJ, Barry SE, Britton WJ, Marks GB. Contact investigation for tuberculosis: a systematic review and meta-analysis. European Respiratory Journal. 2013;41(1):140-56.

[11] Fox J, Weisberg S. An R companion to applied regression. Sage Publications; 2018.

[12] Moran PA. Notes on continuous stochastic phenomena. Biometrika. 1950;37(1/2):17-23.

[13] Li H, Calder CA, Cressie N. Beyond Moran's I: testing for spatial dependence based on the spatial autoregressive model. Geographical Analysis. 2007;39(4):357-75.

[14] Bivand R, Altman M, Anselin L, Assunção R, Berke O, Bernat A, et al. Package 'spdep'. See ftp://garr tucows com/mirrors/CRAN/web/packages/spdep/spdep pdf (accessed 9 December 2015). 2015.
