# Supplementary material for: Spatially-targeted tuberculosis screening has limited impact beyond household contact tracing in Lima, Peru: A model-based analysis
Source: PLoS One. 2023 Oct 30;18(10):e0293519. doi: 10.1371/journal.pone.0293519 (PMC10615320; doi:10.1371/journal.pone.0293519)
Supplement: Peer review history

# Peer review: Decision Letter 0

**DOI:** 10.1371/journal.pone.0293519.r001

**Authors:** Jason M. Haugh, Andrea Ciliberto

20 Apr 2023

Dear Dr. Band,

Thank you very much for submitting your manuscript "Modelling how plant cell-cycle progression leads to cell size regulation" for consideration at PLOS Computational Biology.

As with all papers reviewed by the journal, your manuscript was reviewed by members of the editorial board and by several independent reviewers. In light of the reviews (below this email), we would like to invite the resubmission of a significantly-revised version that takes into account the reviewers' comments.

We cannot make any decision about publication until we have seen the revised manuscript and your response to the reviewers' comments. Your revised manuscript is also likely to be sent to reviewers for further evaluation.

When you are ready to resubmit, please upload the following:

[1] A letter containing a detailed list of your responses to the review comments and a description of the changes you have made in the manuscript. Please note while forming your response, if your article is accepted, you may have the opportunity to make the peer review history publicly available. The record will include editor decision letters (with reviews) and your responses to reviewer comments. If eligible, we will contact you to opt in or out.

[2] Two versions of the revised manuscript: one with either highlights or tracked changes denoting where the text has been changed; the other a clean version (uploaded as the manuscript file).

Important additional instructions are given below your reviewer comments.

Please prepare and submit your revised manuscript within 60 days. If you anticipate any delay, please let us know the expected resubmission date by replying to this email. Please note that revised manuscripts received after the 60-day due date may require evaluation and peer review similar to newly submitted manuscripts.

Thank you again for your submission. We hope that our editorial process has been constructive so far, and we welcome your feedback at any time. Please don't hesitate to contact us if you have any questions or comments.

Sincerely,

Andrea Ciliberto

Academic Editor

PLOS Computational Biology

Jason Haugh

Section Editor

PLOS Computational Biology

***********************

Reviewer's Responses to Questions

**Comments to the Authors:**

**Please note here if the review is uploaded as an attachment.**

Reviewer #1: Balanced growth and division is essential for all forms of life. It is achieved by size control mechanisms operating either before DNA replication or preceding entry into mitosis. It is also important to emphasize that cells do not ‘measure’ their size per se, but rather they are sensing the ratio of cell volume relative to DNA content. Evidence for size control has been well-known for a long time and numerous theoretical models were formulated for explanation. These models broadly speaking fall into one of the following three categories: inhibitor-dilution, activator-accumulation and titration of nuclear sites. Recently, the first two concepts were provided with experimental verification in budding and fission yeast as well as in human cells. In contrast, study of plant cell size control has led to a novel version of titration of nuclear sites model where a cell cycle inhibitor is equally distributed between daughter cells by chromatin binding. All of these background information is provided by this manuscript. The authors develop a comprehensive cell cycle model which shows limit cycle oscillation and it contains two bistable switches operating at G1/S and G2/M transitions. The oscillatory solution of the plant cell cycle control system is impressive. In case all the cell cycle regulators are synthesized in a size-dependent manner the model does not show any size control as expected. Therefore they supplement the model with dilution of size-independent inhibitor or with chromatin mediated equal-inheritance of inhibitor. They find that only combination of two mechanisms provides perfect size control. The following points should be addressed in a revised manuscript.

Major points:

1. The authors should be aware and make it clear that dilution of size-independent inhibitor and chromatin-mediated equal distribution of an inhibitor are not sufficient to provide size control on their own, because both mechanisms require further kinetic constraints and/or assumptions about the expression mechanism. For example size-independent inhibitor-dilution could work without equal-distribution at division if the inhibitor turnovers rapidly And the rate of (amount) synthesis is proportional to the gene copy number. On the other hand, chromatin-mediated equal-inheritance of an inhibitor, as proposed by D’Ario et al., requires the degradation of the unbound fraction of the inhibitor in order to keep the number of inherited molecules proportional to the DNA content.

2. Assuming constant rate of synthesis for size-independent inhibitor implies a rate-limiting step in synthesis, e.g. limiting the transcription rate by gene-copy number. In this case, the synthesis rate cannot constant during the whole cell cycle because if the duplication of the DNA content.

Minor points:

1. The trajectories on the phase plane in Fig.3A and B desperately require arrows to indicate the direction of rotation.

2. On page 20, it is claimed that MYB3R3 (a G2/M CDK inhibitor) cannot provide the basis for size control and conclude 'that this is because MYB3R3 is not directly involved in the hysteretic feedback loop that establishes a bistable switch at G2/M'. It is difficult to follow the authors’ argument, because MYB3R3 is in a double-negative feedback with CDK. The authors should make it clearer why it doesn't contribute to the bistable switch.

3. Fig 9 shows the size homeostasis phenotype of a system with size-independent inhibitor-dilution regulated transitions at G1/S and G2/M. It Is not clear why ‘Birth to G1/S’ has an adder phenotype, while ‘G1/S to division’ has the two-tier sizer/adder phenotype. This may be because the G2/M sizer ensures the birth size is large enough for the G1/S sizer to be cryptic, but the paper makes no comment about it.

Reviewer #2: The paper presents an ODE model for cell cycle and cell size homeostasis in the plant system. ‘In the plant system’ means that the variables and mechanisms at play in the ODEs are taken from the plant literature – as such they will invariably be incomplete (and unlikely to be completely correct), but the attempt is still worthwhile. The analysis is done in a clear straightforward manner. I liked the isolation of the limit cycles for G1/S and G2/M, for example. It also was striking that the equations worked without proposing any cooperativity (no Hill coefficients greater than one), which is somewhat unusual in this sort of paper and suggests a reasonably robust model. In general the model is very carefully explained, and the analysis of how it plays out through multiple cell cycles to give (or not give) cell size homeostasis is done well and explained clearly.

The model is fairly complex in terms of number of variables, and there is a commensurately substantial parameter set. The parameters are (as far as the reader can tell) unconstrained by empirical data, which is always an issue. This is because the literature might propose a general mechanism expressible by an ODE, e.g. ‘KRPs are degraded in response to CDK phosphorylation’ but the functional importance of this claim in the model will depend on the parameters, which will govern how fast this degradation occurs in contrast to baseline degradation, changes in CDK levels (KRP targets) etc. The authors can’t be faulted for not having empirical data for these parameters, but as a fallback it would be useful to try some systematic parameter variations to see how ‘breakable’ the system is. Such an exercise also has the potential to identify the least secure aspects of the model, potentially directing empirical examination of the critical parameter values.

On a related point, many of the conclusions of the paper are based on simulation of results with the current parameter set, which one supposes was optimized initially to get the ‘wild-type’ model to work. So the questions (e.g.) as to whether or to what extent a successful homeostatic model requires both the described G1/S and the G2/M controls might be answered differently with variation of parameter values.

My most general question about the model really is the following: is this truly a plant-specific model, or more a generic model with plant protein names, constrained in a limited way by empirical data? After all, this is a model in the Tyson-Novak vein, of which there have been quite a few published for diverse organisms, but essentially all of which rely critically on bistable states and switches, as does the present model. I realize that the specifics in this model are derived from the plant literature and therefore do not match any of the Tyson-Novak efforts, but I do wonder how much of a translation would be required. Provided the promoters and inhibitors of G1/S and G2/M have the correct signs of activating and inhibiting each other, maybe the plant-specific features of the present model are frequently not critical. Put another way, a model will plausibly require bistable switches to give the expected sort of behavior; but the biochemical mechanisms by which the required mutual inhibition or activation is achieved could be of many different kinds. To take one example, would the current model in which APC is activated by transcription really not work if APC was instead activated by CDK phosphorylation?

To comment generally on the APC: APC almost surely promotes mitosis by other mechanisms, most prominently by degradation of securin (PATRONUS). In yeast and animal systems, APC is activated by cyclin B-CDK, and this is an important feature of control. Another feature that is very important in yeast and animal systems is alternative activation of APC by Cdh1 or by Cdc20 (the former being inhibited by cyclin-CDK, the latter activated). Both CDH1 and CDC20 are conserved in plant genomes; of course, the control circuitry around them might be quite different. So, do we take the present model as making a prediction that cyclin B/Cdc20/Cdh1 control of APC activity doesn't matter in plants? Or that these things are somehow bundled into existing variables and parameters? Or (an unsettling thought) that these things are working in plants and are important there, but the present model can still make an apparently successful simulation while ignoring them altogether?

The paper does not make much of an effort to show how the model does (or doesn’t) accord with available mutant data, nor does it go far to propose predictions (for mutants or physiological perturbations) that might be critical for testing the model. This is, again, an exercise that would distinguish the model as being ‘about’ real plants, as opposed to showing only that a theoretical model of this structure is potentially workable and internally consistent. Please understand, I do not minimize the effort that goes into the latter, but I think after all the Tyson-Novak school of literature, the theoretical contribution here is not adequate to stand on its own; and recently published ODE models do try (to varying degrees) to come to terms with empirical physiological and mutant data.

Minor comments: Ref 15 is cited also as ref 9. The first citation of ref 15 (bottom of p. 2) is really an inadequate exposition of the Whi5 hypothesis. An essential part is that Whi5 is synthesized at a constant amount per cell cycle, independent of cell size. This idea seems to be cited here mostly from the theoretical work in ref 3 (2018), while the idea was originally proposed and supported by strong empirical results in ref 15/9 (2015).

p. 3: ‘functionally conserved’ is not, for me, correct language to suggest the relationship between E2F and SBF or between Rb and Whi5. ‘Conservation’ implies a common ancestry following which some features are retained (‘conserved’). These cases in contrast are almost surely due to convergence of unrelated proteins to carry out similar network roles.

p. 3: to summarize the budding yeast literature as showing they act as ‘adders’ seems like a considerable oversimplification of a lot of literature, showing a strong though imperfect sizing component to the budding yeast G1/S control. This includes the Whi5 story that has already been cited. (This literature goes back at least to the early 1980’s [e.g. Work by Alan Wheals on 'sloppy size control'] and is still an area of active work. I understand this paper is not a review of the literature but this goes too far in the other direction).

Reviewer #3: Manuscript entitled „Modelling how plant cell-cycle progression leads to cell size regulation” dealing with different cell size controlling mechanisms operating at the two major cell cycle transitions G1-S and G2-M in plants. Authors are experts on this field as they have already published excellent results focusing on cell size control functioning during plant cell cycle (eg. Jones et al., 2017; 2019). Here on the basis of mathematical models they do simulations in cell populations, and suggested integrating size-control mechanisms at both G1/S and G2/M is essential to maintain cell-size homeostasis.

Recent new results emerging from this field including the equal inheritance of KRP4 at G1-S (D’Ario et al., 2021) and the role of SMRs in the control of G2-M (Nomoto et al., 2022) indicating that both transitions in cycling cells could serve as size controller. The authors built these new cell size-mechanisms into their simulations, and resulted in an interesting model. One of their major findings is that equal inheritance of KRP, a plant specific CDK inhibitor functioning in G1 could not alone be itself a „sizer” but in combination with a G2 CDK inhibitor SMR protein. This model might explain why plant cells specifically control their size at two major cell cycle transition points.

In yeast and probably in animal cells as well, an increasing CDK activity drives unidirectional cell cycle progression to one phase to another and reaching its maximum in M-phase. This look different in plant cells as two peaks of CDK activity was detected at the major transition points (at G1/S and G2/M, respectively). KRPs and SMRs are the inhibitors of different CDKs like the types of CDKA and CDKB. Why do plant cells have these unique regulations? How do they control/fine tune each other? These are rather theoretical questions.

Minor points.

Figure 1. Neither dashed nor dotted lines in their model.

Figure 3. I am not sure that experimental data supporting all these simulations: Do really the concentrations of CDKA:CYCD and CDKB:CYCB complexes peak at the same time in the G2 phase? E2FA and E2FB were also shown to have different cell cycle dependency as E2FA peaks in S while E2FB is more constitutive during cell cycle. Concentration of RBR was also shown to be unchanged throughout cell cycle but supposed to be its phosphorylation level (and consequently its activity) changes.

Figure 5. Why SMOS1:SCL28 mentioned in the legend? Why they are not presented in the graph? Some introductions require.

On top of page 27: “Cyclin B inhibiting transcription factor, SMR, is size independent” SMR is a CDK inhibitor.

**********

**Have the authors made all data and (if applicable) computational code underlying the findings in their manuscript fully available?**

The PLOS Data policy requires authors to make all data and code underlying the findings described in their manuscript fully available without restriction, with rare exception (please refer to the Data Availability Statement in the manuscript PDF file). The data and code should be provided as part of the manuscript or its supporting information, or deposited to a public repository. For example, in addition to summary statistics, the data points behind means, medians and variance measures should be available. If there are restrictions on publicly sharing data or code —e.g. participant privacy or use of data from a third party—those must be specified.

Reviewer #1: Yes

Reviewer #2: Yes

Reviewer #3: Yes

**********

PLOS authors have the option to publish the peer review history of their article (what does this mean?). If published, this will include your full peer review and any attached files.

If you choose “no”, your identity will remain anonymous but your review may still be made public.

**Do you want your identity to be public for this peer review?** For information about this choice, including consent withdrawal, please see our Privacy Policy.

Reviewer #1: No

Reviewer #2: No

Reviewer #3: No

Figure Files:

While revising your submission, please upload your figure files to the Preflight Analysis and Conversion Engine (PACE) digital diagnostic tool, https://pacev2.apexcovantage.com. PACE helps ensure that figures meet PLOS requirements. To use PACE, you must first register as a user. Then, login and navigate to the UPLOAD tab, where you will find detailed instructions on how to use the tool. If you encounter any issues or have any questions when using PACE, please email us at figures@plos.org.

Data Requirements:

Please note that, as a condition of publication, PLOS' data policy requires that you make available all data used to draw the conclusions outlined in your manuscript. Data must be deposited in an appropriate repository, included within the body of the manuscript, or uploaded as supporting information. This includes all numerical values that were used to generate graphs, histograms etc.. For an example in PLOS Biology see here: http://www.plosbiology.org/article/info%3Adoi%2F10.1371%2Fjournal.pbio.1001908#s5.

Reproducibility:

To enhance the reproducibility of your results, we recommend that you deposit your laboratory protocols in protocols.io, where a protocol can be assigned its own identifier (DOI) such that it can be cited independently in the future. Additionally, PLOS ONE offers an option to publish peer-reviewed clinical study protocols. Read more information on sharing protocols at https://plos.org/protocols?utm_medium=editorial-email&utm_source=authorletters&utm_campaign=protocols

# Author comment: Author response to Decision Letter 0

**DOI:** 10.1371/journal.pone.0293519.r002

26 Jul 2023

Attachment Submitted filename: Plant cell cycle modelling - response to reviewers-4.pdf Click here for additional data file.

# Peer review: Decision Letter 1

**DOI:** 10.1371/journal.pone.0293519.r003

**Authors:** Jason M. Haugh, Andrea Ciliberto

7 Sep 2023

Dear Dr. Band,

We are pleased to inform you that your manuscript 'Modelling how plant cell-cycle progression leads to cell size regulation' has been provisionally accepted for publication in PLOS Computational Biology.

Before your manuscript can be formally accepted you will need to complete some formatting changes, which you will receive in a follow up email. A member of our team will be in touch with a set of requests.

Please note that your manuscript will not be scheduled for publication until you have made the required changes, so a swift response is appreciated.

IMPORTANT: The editorial review process is now complete. PLOS will only permit corrections to spelling, formatting or significant scientific errors from this point onwards. Requests for major changes, or any which affect the scientific understanding of your work, will cause delays to the publication date of your manuscript.

Should you, your institution's press office or the journal office choose to press release your paper, you will automatically be opted out of early publication. We ask that you notify us now if you or your institution is planning to press release the article. All press must be co-ordinated with PLOS.

Thank you again for supporting Open Access publishing; we are looking forward to publishing your work in PLOS Computational Biology. 

Best regards,

Andrea Ciliberto

Academic Editor

PLOS Computational Biology

Jason Haugh

Section Editor

PLOS Computational Biology

***********************************************************

Reviewer's Responses to Questions

**Comments to the Authors:**

**Please note here if the review is uploaded as an attachment.**

Reviewer #1: I am happy with the revision except the size of the file (>45MB)

Reviewer #3: I do not have further questions.

**********

**Have the authors made all data and (if applicable) computational code underlying the findings in their manuscript fully available?**

The PLOS Data policy requires authors to make all data and code underlying the findings described in their manuscript fully available without restriction, with rare exception (please refer to the Data Availability Statement in the manuscript PDF file). The data and code should be provided as part of the manuscript or its supporting information, or deposited to a public repository. For example, in addition to summary statistics, the data points behind means, medians and variance measures should be available. If there are restrictions on publicly sharing data or code —e.g. participant privacy or use of data from a third party—those must be specified.

Reviewer #1: Yes

Reviewer #3: Yes

**********

PLOS authors have the option to publish the peer review history of their article (what does this mean?). If published, this will include your full peer review and any attached files.

If you choose “no”, your identity will remain anonymous but your review may still be made public.

**Do you want your identity to be public for this peer review?** For information about this choice, including consent withdrawal, please see our Privacy Policy.

Reviewer #1: No

Reviewer #3: No

# Editor report: Acceptance letter

**DOI:** 10.1371/journal.pone.0293519.r004

**Authors:** Jason M. Haugh, Andrea Ciliberto

4 Oct 2023

PCOMPBIOL-D-23-00356R1 

Modelling how plant cell-cycle progression leads to cell size regulation

Dear Dr Band,

I am pleased to inform you that your manuscript has been formally accepted for publication in PLOS Computational Biology. Your manuscript is now with our production department and you will be notified of the publication date in due course.

The corresponding author will soon be receiving a typeset proof for review, to ensure errors have not been introduced during production. Please review the PDF proof of your manuscript carefully, as this is the last chance to correct any errors. Please note that major changes, or those which affect the scientific understanding of the work, will likely cause delays to the publication date of your manuscript. 

Soon after your final files are uploaded, unless you have opted out, the early version of your manuscript will be published online. The date of the early version will be your article's publication date. The final article will be published to the same URL, and all versions of the paper will be accessible to readers.

Thank you again for supporting PLOS Computational Biology and open-access publishing. We are looking forward to publishing your work! 

With kind regards,

Timea Kemeri-Szekernyes

PLOS Computational Biology | Carlyle House, Carlyle Road, Cambridge CB4 3DN | United Kingdom ploscompbiol@plos.org | Phone +44 (0) 1223-442824 | ploscompbiol.org | @PLOSCompBiol
